# Modulation of pulmonary desmosomes by inhaler therapy in preterm-born children with bronchopulmonary dysplasia

**DOI:** 10.1038/s41598-023-34233-5

**Published:** 2023-05-05

**Authors:** Christopher W. Course, Philip A. Lewis, Sarah J. Kotecha, Michael Cousins, Kylie Hart, W. John Watkins, Kate J. Heesom, Sailesh Kotecha

**Affiliations:** 1 Department of Child Health, School of Medicine, Cardiff University, Cardiff, CF14 4XN UK; 2 Proteomics Facility, Faculty of Life Sciences, University of Bristol, Bristol, UK; 3 Department of Paediatrics, Cardiff and Vale University Health Board, Cardiff, UK

**Keywords:** Proteomics, Respiratory tract diseases, Paediatrics, Preterm birth

## Abstract

Despite evidence demonstrating persistent lung function deficits in preterm-born children, especially in those who had bronchopulmonary dysplasia (BPD) in infancy, the underlying biological mechanisms explaining these lung function deficits remain poorly understood. We characterised the exhaled breath condensate (EBC) proteome in preterm-born children, with and without BPD; and before and after inhaler treatment. EBC from children aged 7–12 years, from the Respiratory Health Outcomes in Neonates (RHiNO) study, were analysed by Nano-LC Mass Spectrometry with Tandem Mass Tag labelling. Children with percent predicted forced expiratory volume in 1 second ≤ 85% were enrolled to a 12-week blinded randomised trial of inhaled corticosteroids alone (ICS) or with long-acting β_2_-agonist (ICS/LABA) or placebo. EBC was analysed from 218 children at baseline, and 46 children received randomised inhaled therapy. 210 proteins were detected in total. For the 19 proteins present in every sample, the desmosome proteins: desmoglein-1, desmocollin-1 and plakoglobin were significantly decreased, and cytokeratin-6A was increased in preterm-born children with BPD when compared to preterm- and term-born controls. ICS/LABA treatment significantly increased abundance of desmoglein-1, desmocollin-1 and plakoglobin in the BPD group with low lung function, and significantly increased plakoglobin in those without BPD. No differences were noted after ICS treatment. Exploratory analyses of proteins not detected in all samples suggested decreased abundance of several antiproteases. This study provides proteomic evidence of ongoing pulmonary structural changes with decreased desmosomes in school-aged preterm-born children with BPD and low lung function, which was reversed with combined inhaled corticosteroids and long-acting β_2_-agonists therapy.

## Introduction

Respiratory pathophysiology is a common long-term consequence of preterm birth^1^ and Bronchopulmonary Dysplasia (BPD), also called Chronic Lung Disease of Prematurity, remains a major clinical challenge for neonatologists^2^. It is well-established that survivors of preterm birth, both with and without a diagnosis of BPD, are at risk of persistent lung function deficits throughout childhood and adulthood^3^ with increased propensity to respiratory symptoms, increased hospitalisation and increased inhaler use^4^, with potential premature development of chronic obstructive pulmonary disease (COPD)^5^. Whilst many of these children are diagnosed with asthma, it is becoming apparent that there are more complex respiratory phenotypes following preterm birth^6,7^; however, the underlying pathophysiology remains poorly characterised^8^. Our recent randomised controlled trial (RCT) showed that combined inhaled corticosteroids and long acting β_2_-agonists was an effective treatment for prematurity-associated lung disease^7^, but only by understanding the underlying mechanisms, to identify the various underlying endotypes, can appropriate therapeutic interventions be developed and brought into clinical use.

Exhaled breath condensate (EBC) provides a useful sample to study in children due to its ease in collection. EBC is composed of droplets of the airway lining fluid (ALF), evolved from all compartments of the lung during tidal breathing. It is a complex mixture of DNA, RNA, proteins, metabolites and volatile organic compounds reflecting lung tissue biology^9^. EBC is of interest for studying respiratory pathology due to its simple, non-invasive, and easily repeatable method of collection^10^ and has been used to study mechanisms in asthma, COPD and bronchiolitis. Identifying the proteome in EBC has been challenging, but recent developments aid identification and accurate quantification of large arrays of proteins. Proteomics methods simultaneously analyse the entire protein complement of biological samples and have gained interest clinically as a potential tool for unravelling disease pathogenesis and identifying biomarkers^11–13^.

We hypothesised that the EBC proteome in preterm-born children with lung disease would be altered when compared to term born controls; and that treatment, in an RCT, would normalise any abnormalities detected. We, therefore, compared the EBC proteome obtained from preterm-born school-aged children, with and without BPD, to term-born controls. In addition, we compared the EBC proteome in preterm-born children with low lung function, who were treated with inhaled corticosteroids (ICS), a combination of ICS and long-acting β2 agonist (LABA) and placebo in a RCT^7^.

## Methods

### Participants

This study was conducted on a cohort of children recruited to the Respiratory Health Outcomes in Neonates study (RHiNO, EudraCT: 2015-003712-20) which has been described previously^6,7^. Briefly, children from a previous study^4^ were supplemented with additional preterm-born children identified by the NHS Wales Informatics Service and sent a respiratory and neurodevelopmental questionnaire if they were born ≤ 34 or ≥ 37 weeks’ gestation and were aged 7–12 years. Children with significant congenital malformations, cardiopulmonary or neuromuscular disease were excluded. Ethical approval was obtained from the South-West Bristol Research Ethics Committee (15/SW/0289). Parents gave informed written consent and children provided assent. The study was conducted according to the Good Clinical Practice (GCP) guidelines and the Declaration of Helsinki.

Responders were assessed at their home and a subset attended the hospital children’s research facility for comprehensive respiratory testing including collection of EBC (RTube®, Respiratory Research Inc. Texas, USA), conducted by a trained nurse and paediatrician between January 2017 and November 2019. Spirometry (MasterScreen Body and PFT systems, Vyaire Medical, Germany) was performed to ATS/ERS guidelines^14^ and normalised using Global Lung Initiative (GLI) references^15^. Those preterm-born children with low lung function (PT_low_) defined as percent predicted forced expiratory volume in 1 second (%FEV_1_) of ≤ 85% were enrolled into the RCT^7^. Term-born children who had %FEV_1_ > 90% were included as term controls. BPD was defined as oxygen-dependency of 28 days or greater for those born < 32 weeks’ gestation and at 56 days of age for those born ≥ 32 weeks’ gestation)^16^. Intrauterine growth restriction (IUGR) was defined as birthweight < 10th percentile adjusted for sex and gestation (LMS Growth version 2.77, Medical Research Council, UK). Neonatal history was corroborated with medical records.

PT_low_ participants were enrolled to a twelve-week blinded RCT, receiving ICS (50 μg fluticasone propionate), a combination of ICS and LABA (ICS/LABA) (50 μg fluticasone propionate and 25 μg salmeterol xinafoate) or placebo. Following treatment, RCT participants underwent repeat EBC sampling. The RCT has recently been published^7^ and is described in detail in the online supplement.

### EBC sampling

EBC was collected in a standardised manner using a cooling tube (RTube®, Respiratory Research Inc. Texas, USA) over a period of 10 min of passive tidal breathing whilst the participant wore a nose clip, stopping briefly to swallow saliva if needed, as per manufacturer’s guidance. The RTube® is a single-patient, single-use design, preventing cross contamination, and features a large ‘Tee’ section to separate saliva from exhaled breath, thereby ensuring collection of airway lining fluid and not secretions from the oropharynx. Once collected, samples were immediately separated into aliquots and stored at − 70 °C pending analysis.

### Sample analysis

In brief, 100 µL of each sample (ensuring that no sample contained more than 50 µg of protein) was digested with trypsin, labelled with Tandem Mass Tag (TMT) eleven plex reagents (Thermo Fisher Scientific, Loughborough, UK) and the labelled samples pooled. The TMT-labelled pool was fractionated using an Ultimate 3000 nano-LC system (Thermo Scientific). All spectra were acquired using an Orbitrap Fusion Lumos mass spectrometer (Thermo Scientific) controlled by Xcalibur 3.0 software (Thermo Scientific) and operated in data-dependent acquisition mode using an SPS-MS3 workflow. The raw data files were processed and quantified using Proteome Discoverer software v2.1 (Thermo Scientific) and searched against the UniProt Human database (downloaded October 2019: 150,786 entries). Further detail on sample processing and proteomic analysis are described in the online supplement.

### Statistical analysis

Baseline population and RCT group characteristics were compared using Chi-squared, t-test or one-way ANOVA with Bonferroni correction as appropriate. Replicate numbers (number of samples in which a particular protein was detected) were calculated. Relative protein abundances, determined from the quantity of TMT-tag counts at each detected peptides spectral peak, were log_2_-transformed and fold changes (log_2_FC) between groups were compared, and the data inspected for normality. Welch’s t-test/ANOVA with post-hoc Tukey correction was used for baseline samples as appropriate, and paired samples t-test for pre/post-RCT samples. p < 0.05 was considered statistically significant. All analyses were performed using R v4.0.4^17^. Gene name is used synonymously with protein name. Gene names were unavailable for four proteins. WebGestalt was used to perform functional enrichment analysis^18^. Ingenuity Pathways Analysis (IPA, Qiagen®, Germany) identified relationships between significantly different proteins using network maps, which were reproduced for publication in Cytoscape v3.9^19^. Linear regression models were used to identify associations between participant characteristics and proteins of interest.

## Results

From 1426 returned questionnaires, 768 children had home assessments and 241, including 53 enrolling into the RCT, underwent detailed assessments. EBC was successfully collected and analysed from 218 (91%) children at baseline. 48 of the 53 RCT participants completed treatment and 46/48 (96%) post-treatment EBC samples were successfully collected and analysed. Participant demographics at baseline and for the RCT groups are shown in Table 1. At baseline, significant differences were noted between the preterm-born and term-born children for age at testing (mean 11.01 ± 1.24 years vs 10.43 ± 1.09, p = 0.001) and asthma diagnosis (34 (23%) vs 5 (7%), p = 0.007). Thirty-seven (25%) of the preterm-born children had a neonatal diagnosis of BPD and 53 (36%) were classed PT_low_, all of whom joined the RCT. There were no differences for asthma diagnosis (10 (27%) vs 24 (21%); p = 0.67) or IUGR (8 (22%) vs 19 (17%); p = 0.70) between the preterm BPD and No BPD groups, nor for between the PT_low_ and PT_c_ groups (asthma: 16 (30%) vs 18 (19%); p = 0.19; IUGR: 12 (23%) vs 15 (16%); p = 0.35 respectively). Marginally more EBC was collected from term-born children compared to preterm-born (1.13 ml vs 1.28 ml, p = 0.001), but no significant differences were noted between the preterm groups (BPD or PT_low_ vs preterm controls, p = 1.0]) or between the three RCT groups. Demographics were similar for the three RCT groups. However, the placebo group produced more EBC after treatment (p = 0.02), but not for ICS or ICS/LABA (p > 0.1).

**Table 1:** Participant demographics.

Baseline			
Variable	Preterm born ($\le$ 34/40) n = 149	Term born ($\ge$ 37/40) n = 69	
Sex (male), n(%)	71 (48)	36 (52)	
Ethnicity (white), n(%)	140 (94)	68 (99)	
Gestational age (weeks), mean (SD)	30.90 (2.75)	40.19 (1.10)***	
Birthweight (g), mean (SD)	1613 (587)	3521 (518)***	
Intrauterine growth restriction, n(%)	27 (18)	4 (6)*	
Bronchopulmonary dysplasia, n(%)	37 (25)	0 (0)***	
Age at testing (years), mean (SD)	11.01 (1.24)	10.43 (1.09)**	
Weight (kg), mean (SD)	39.07 (10.70)	37.89 (10.52)	
Body Mass Index (kg/m^2^), mean (SD)	18.21 (3.46)	18.04 (3.15)	
Asthma diagnosis, n(%)	34 (23)	5 (7)**	
Low lung function (FEV_1_ $\le$ 85%pred), n(%)	53 (36)	0 (0)	
Total volume of EBC collected (ml), mean (SD)	1.13 (0.29)	1.28 (0.31)**	

Post RCT samples			
Variable	Preterm born ($\le$ 34/40) with low lung function (FEV_1_ $\le$ 85%) n = 46		
	Placebo n = 12	ICS n = 17	ICS/LABA n = 17
Sex (male), n(%)	5 (39)	6 (35)	8 (47)
Ethnicity (white), n(%)	13 (100)	14 (82)	17 (100)
Gestational age (weeks), mean (SD)	29.61 (3.26)	29.40 (2.97)	30.86 (2.83)
Birthweight (g), mean (SD)	1394 (612)	1282 (545)	1470 (570)
Intrauterine growth restriction, n(%)	2 (17)	2 (12)	6 (35)
Bronchopulmonary dysplasia, n(%)	7 (54)	7 (41)	6 (35)
Age at testing (years), mean (SD)	11.04 (1.21)	10.68 (1.36)	10.69 (1.23)
Weight (kg), mean (SD)	38.89 (11.26)	36.66 (12.38)	36.47 (9.67)
Body Mass Index (kg/m^2^), mean (SD)	18.03 (3.61)	17.87 (3.86)	17.35 (2.31)
Asthma diagnosis, n(%)	2 (17)	8 (47)	4 (24)
Total volume of EBC collected pre-treatment (ml), mean (SD)	1.08 (0.32)	1.23 (0.28)	1.09 (0.26)
Total volume of EBC collected post-treatment (ml), mean (SD)	1.33 (0.33)^ƚ^	1.31 (0.20)	1.22 (0.26)

Preterm born vs Term born: *p < 0.05, **p < 0.01 ***p < 0.001 Chi-squared test/independent samples t-test as appropriate.

Treatment groups: *p < 0.05, **p < 0.01 ***p < 0.001 Chi-squared test/ANOVA with Bonferroni correction as appropriate.

Comparing EBC volumes within groups pre and post treatment: ^ƚ^p < 0.05 by paired samples t-test.

EBC, exhaled breath condensate; ICS, inhaled corticosteroids; ICS/LABA, inhaled corticosteroids and long-acting beta agonist. No significant differences seen between RCT treatment groups.

We identified 210 different proteins as detailed in the online supplementary Table 1 together with replicate number. The distribution of detected proteins across all samples is shown in the heatmap (Supplementary Fig. 1). Functional enrichment analysis (which determines classes of proteins that are over-represented within a large group of proteins) was possible for 192 proteins (Supplementary Fig. 2). Twenty-eight proteins were identified with a significant difference between one or more of the group comparisons and functional enrichment analysis was possible for 27 of these. Most proteins with significantly differing abundances were functionally related to protein/ion binding and cell structure.

### Baseline samples

Nineteen proteins were detected in all 218 baseline EBC samples (Table 2). Cytokeratins were the most detected protein class. Increased abundance of two keratins, type II cytoskeletal 5 (KRT5) (0.12, p = 0.03) and 6A (KRT6A) (0.14, p = 0.02) was observed when the all preterm-born and term-born groups were compared.

**Table 2:** Proteins detected in every sample.

UniProt accession number	Gene name	Protein name	Protein function	Preterm vs Term n = 149 vs 69			BPD vs No BPD n = 37 vs 112			PT_low_ vs PT_c_ n = 53 vs 96		
				Log_2_FC	Ratio	p	Log_2_FC	Ratio	p	Log_2_FC	Ratio	p
Q08554	DSC1	Desmocollin-1	Cell–cell junction	0.02	1.01	0.84	**− 0.27**	**0.83**	0.02*	− 0.08	0.95	0.43
Q02413	DSG1	Desmoglein-1	Cell–cell junction	**− 0.17**	**0.89**	0.03*	**− 0.26**	**0.84**	0.02*	− 0.16	0.90	0.10
P15924	DSP	Desmoplakin	Cell–cell junction, Cytoskeleton	− 0.005	0.997	0.94	− 0.08	0.95	0.35	0.11	1.08	0.13
P14923	JUP	Junction plakoglobin	Plasma membrane protein complex	− 0.08	0.95	0.30	− **0.23**	**0.85**	0.04*	− 0.10	0.93	0.27
H6VRG2	KRT1	Cytokeratin-1	Cytoskeleton	0.06	1.04	0.45	− 0.15	0.90	0.13	− 0.04	0.97	0.62
H6VRG3	KRT1	Cytokeratin-1	Cytoskeleton	0.13	1.09	0.24	− 0.06	0.96	0.68	− 0.01	0.99	0.94
P35908	KRT2	Keratin, type II cytoskeletal 2 epidermal	Cytoskeleton	− 0.18	0.88	0.06	0.11	1.08	0.45	0.06	1.04	0.64
P13647	KRT5	Keratin, type II cytoskeletal 5	Cytoskeleton	**0.12**	**1.09**	0.03*	0.13	1.09	0.14	0.05	1.04	0.52
P02538	KRT6A	Keratin, type II cytoskeletal 6A	Cytoskeleton	**0.41**	**1.33**	0.02*	**0.68**	**1.60**	0.01*	0.14	1.10	0.55
P35527	KRT9	Keratin, type I cytoskeletal 9	Cytoskeleton	0.19	1.14	0.06	− 0.20	0.87	0.08	− 0.09	0.94	0.38
P13645	KRT10	Keratin, type I cytoskeletal 10	Structural protein extracellular space	− 0.08	0.95	0.39	0.07	1.05	0.49	0.04	1.03	0.64
P02533	KRT14	Keratin, type I cytoskeletal 14	Cytoskeleton	− 0.006	0.996	0.91	0.14	1.10	0.14	0.06	1.04	0.45
P08779	KRT16	Keratin, type I cytoskeletal 16	Cytoskeleton	0.31	1.24	0.10	0.40	1.32	0.17	0.28	1.21	0.25
Q04695	KRT17	Keratin, type I cytoskeletal 17	Intermediate filament cytoskeleton	− 0.04	0.97	0.79	0.36	1.28	0.09	0.10	1.07	0.58
Q8N1N4	KRT78	Keratin, type II cytoskeletal 78	Cytoskeleton	0.03	1.02	0.73	0.07	1.05	0.44	− 0.02	0.99	0.86
P31944	CASP14	Caspase-14	Protease	0.13	1.09	0.4	0.01	1.01	0.95	0.11	1.08	0.62
P01040	CSTA	Cystatin-A	Protease inhibitor	0.13	1.09	0.27	− 0.19	0.88	0.22	0.05	1.04	0.72
P62979	RPS27A	Ubiquitin-40S ribosomal protein S27a	Structural component of ribosome	0.09	1.06	0.41	− 0.31	0.81	0.06	0.30	1.23	0.05
P25311	AZGP1	Zinc-alpha-2-glycoprotein	Major histocompatibility complex protein	− 0.02	0.99	0.86	− 0.27	0.83	0.11	0.18	1.13	0.27

BPD, preterm-born with history of bronchopulmonary dysplasia; PT_c_, preterm-born control; PT_low_, preterm born with low lung function; Log_2_FC, Log_2_ fold-change between groups. *Denotes p-vale < 0.05 from independent samples t-test.

Significant values are in [bold].

**Figure 1:**
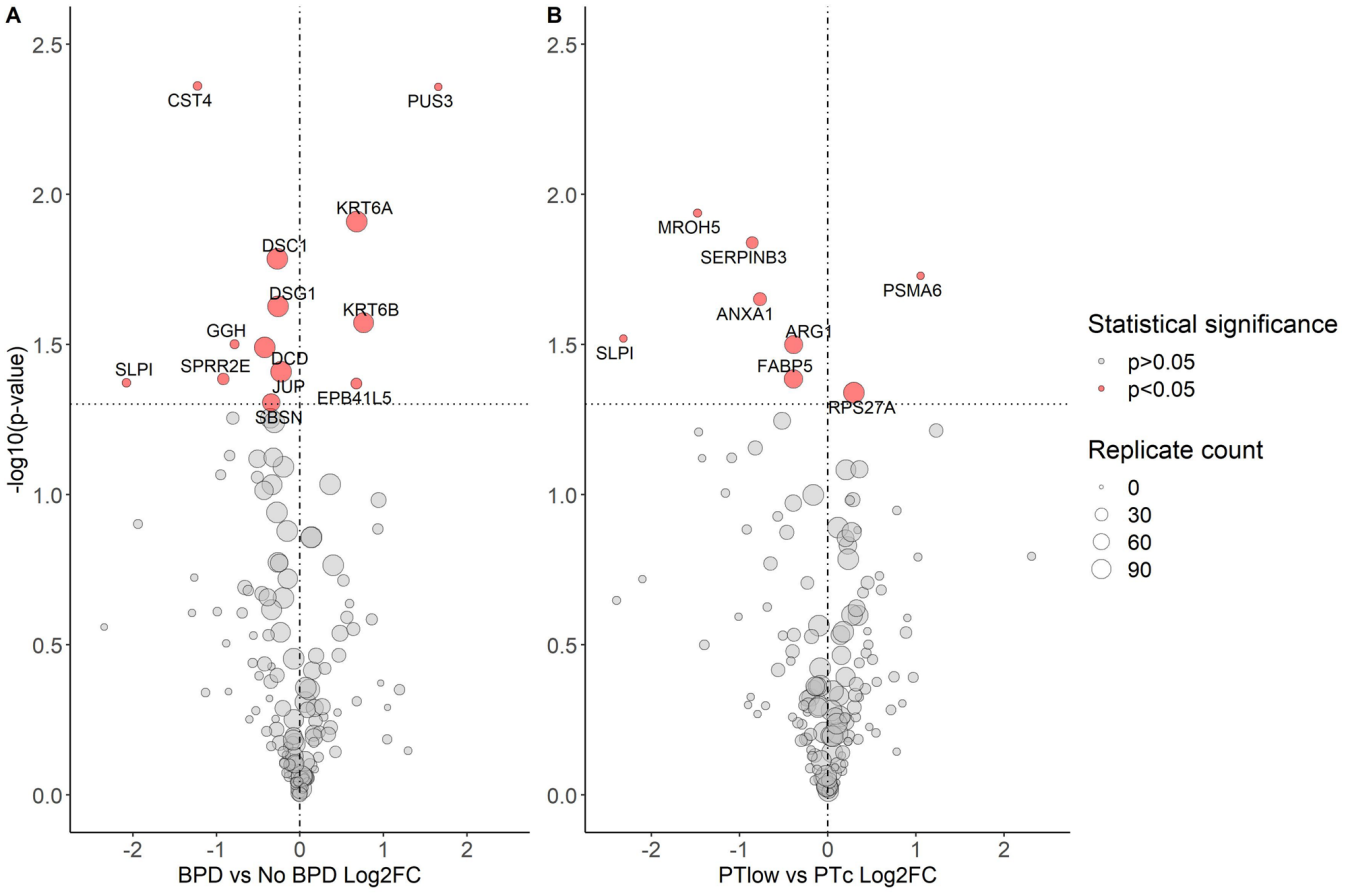
Volcano Plots demonstrating baseline protein abundance by BPD and Lung Function Status for Preterm-born children. Vertical line represents a Log_2_FC of 0. Horizontal line is equivalent to p-value 0.05. Size of point is relative to number of samples in which protein was detected. Gene name associated with protein given if p < 0.05. BPD, bronchopulmonary dysplasia; PTlow, preterm-born with low lung function; PTc, preterm-born control; Log2FC, Log_2_ fold-change between groups.

Exploratory analyses of proteins with a significant abundance difference between the groups that were not detected in every sample are shown in Supplementary Table 2, ordered by decreasing replicate number. Eleven proteins were detected with significant differences between preterm- and term-born children, nine between BPD and No BPD, and seven between PT_low_ and PT_c_ groups. Figure 1 shows all significantly different protein abundances between the BPD and No BPD groups; and PT_low_ and PT_c_ groups.

#### BPD vs No BPD

**Figure 2:**
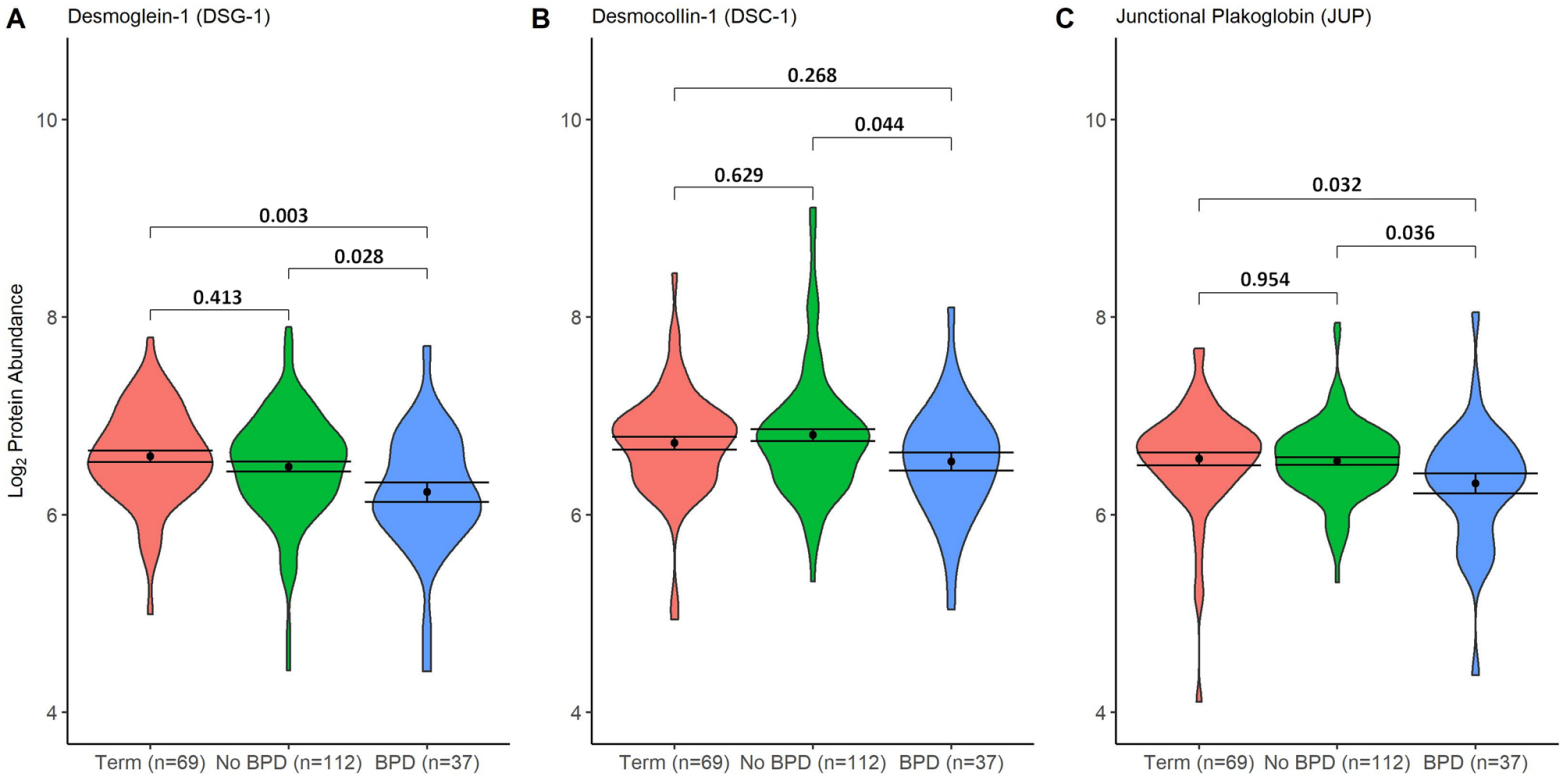
Violin plots: Desmosome/cell adhesion baseline protein abundances in children with history of BPD. Term, term-born control; No BPD, preterm-born without BPD; BPD, bronchopulmonary dysplasia; Dot and bars represent mean and standard error (SEM); Comparison bars between violin plots give p-values by ANOVA with post-hoc Tukey’s for multiple comparisons. Coloured areas represent distribution of sample values.

For proteins detected in every sample, significantly decreased abundance (determined by TMT tag count at the spectral peak) of the desmosome proteins desmoglein-1 (DSG1) (Log_2_FC − 0.26, p = 0.02), desmocollin-1 (DSC1) (− 0.27, p = 0.02) and junctional plakoglobin (JUP) (− 0.23, p = 0.04), and increased abundance of KRT6A (0.68, p = 0.01) was observed when the BPD and No BPD groups were compared (Table 2). No significant differences were noted for DSG1, DSC1 and JUP between the No BPD and Term groups (Fig. 2).

**Figure 3:**
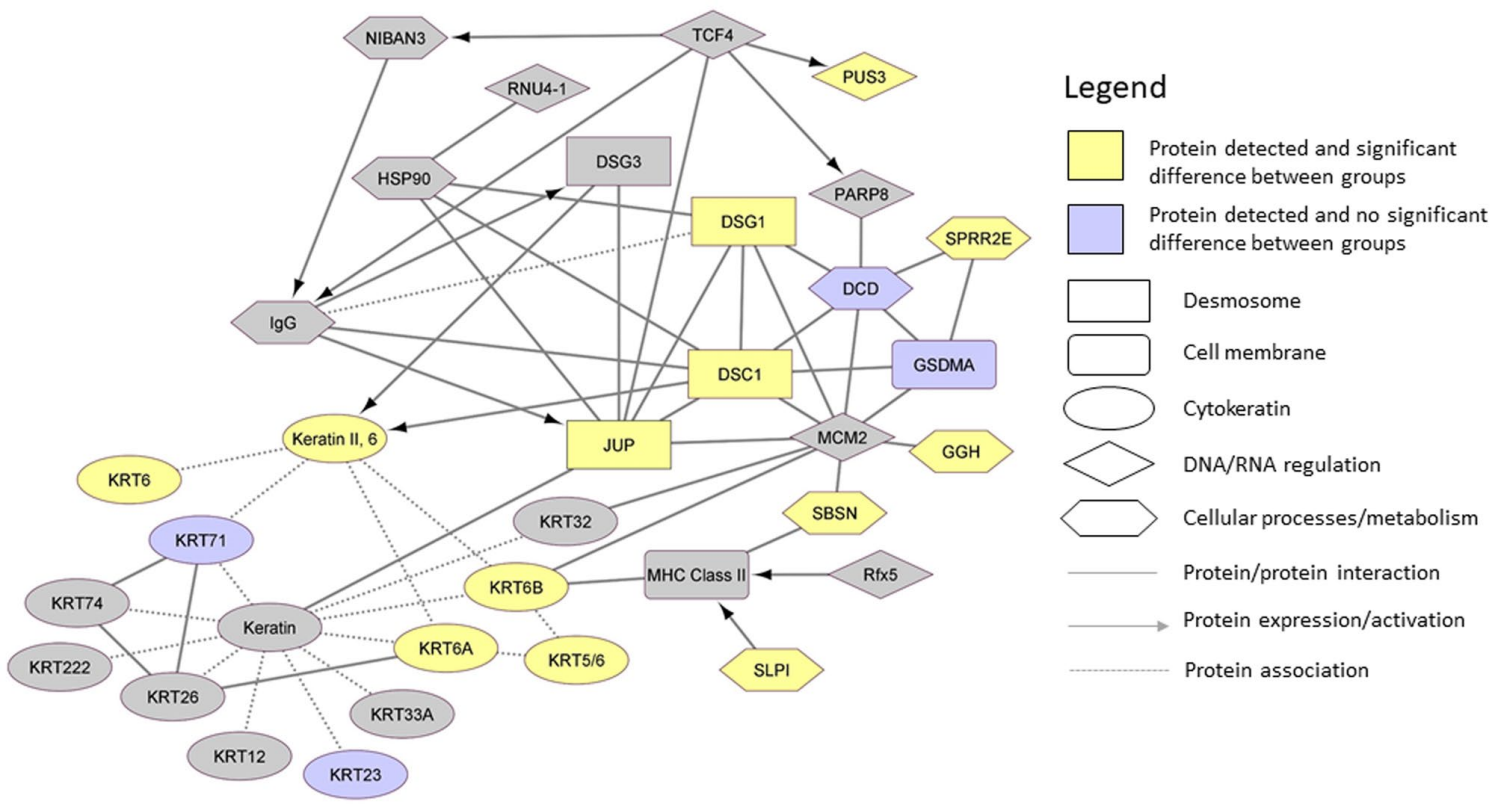
Protein network map of significant protein differences between BPD and No BPD Preterm-born children.

Protein network maps highlighting significant protein pathways (including proteins detected in all or only some samples) comparing BPD and No BPD groups are shown in Fig. 3. For proteins detected in a proportion of samples, dermcidin (DCD) was detected in n = 146 (98%) samples and was less abundant in the BPD group (Log_2_FC − 0.43, p = 0.03), and was related to DSG1 and DSC1 in the network map. As with KRT6A, KRT6B was detected in 133 (89%) samples, being more abundant in the BPD group (0.76, p = 0.03) when compared to the No BPD group. Small proline-rich protein 2E (SPRR2E), secretory leukocyte peptidase inhibitor (SLPI) and gamma-glutamyl hydrolase (GGH) were all significantly less abundant in the BPD group (− 0.92, p = 0.04; − 2.08, p = 0.04; − 0.79, p = 0.03 respectively); however, these were detected in < 25% of the samples.

Univariable linear regression analyses for DSG1, DSC1 and JUP (Table 3) identified that a history of BPD had significant association with each of these three proteins (β−0.23, p = 0.014; β−0.27, p = 0.019; β−0.23, p = 0.008 respectively) but sex, age, diagnosis of asthma and low lung function did not. Since low lung function in those with BPD was associated with lower desmosome proteins, we assessed the interaction of BPD and low lung function: the results showed that the reduced abundance of DSG1, DSC1 and JUP was significantly or near significantly associated with those with BPD and PT_low_ (β−0.35, p = 0.012; β−0.30, p = 0.06, β−0.30, p = 0.01 respectively) but not in the BPD group who had normal lung function (Table 3).

**Table 3:** Linear regression analyses in preterm-born school-aged children.

Univariable analysis						
	Desmoglein-1 (DSG1)		Desmocollin-1 (DSC1)		Junctional plakoglobin (JUP)	
Variable	Beta	p-value	Beta	p-value	Beta	p-value
Sex (Ref = Male)	− 0.10	0.26	0.07	0.52	− 0.02	0.77
Age	− 0.04	0.34	-0.03	0.45	− 0.06	*0.06*
BPD (Ref = No BPD)	− 0.23	0.014*	− 0.27	0.019*	− 0.23	0.008*
Low Lung Function (Ref = PT_c_)	− 0.16	0.10	− 0.09	0.43	− 0.10	0.21
Asthma (Ref = No)	− 0.05	0.66	− 0.09	0.49	− 0.15	*0.09 *

Interaction modelling						
	DSG1		DSC1		JUP	
	Beta	p-value	Beta	p-value	Beta	p-value
No BPD * PT_c_	Ref	Ref	Ref	Ref	Ref	Ref
No BPD * PT_low_	− 0.12	0.30	− 0.04	0.73	− 0.04	0.66
BPD * PT_c_	− 0.23	0.11	− 0.27	*0.09*	− 0.18	0.12
BPD * PT_low_	− 0.35	0.012*	− 0.30	*0.06*	− 0.30	0.01*

Significant values are in bold.

*p < 0.05, italic = p < 0.1. BPD, bronchopulmonary dysplasia; PT_low_, preterm-born low lung function; PT_c_, preterm-born controls.

#### PT_low_ vs PT_c_

For proteins detected in all samples, no significant differences were noted between the PT_low_ group and PT_c_ groups at baseline. A protein network map including all detected proteins with significant differences between PT_low_ and PT_c_ groups is given in Supplementary Fig. 3. Three antiproteases (Annexin A1 [ANXA1], Serpin B3 [SERPINB3], SLPI) were less abundant in the PT_low_ group (− 0.77, p = 0.02; − 0.86, p = 0.01; − 2.32, p = 0.03 respectively), with reduced abundance of fatty acid-binding protein 5 (FABP5) (− 0.39, p = 0.04) when compared to the PT_c_ group. The network map (Supplementary Fig. 3) did not demonstrate any direct links between these proteins.

#### RCT group

**Figure 4:**
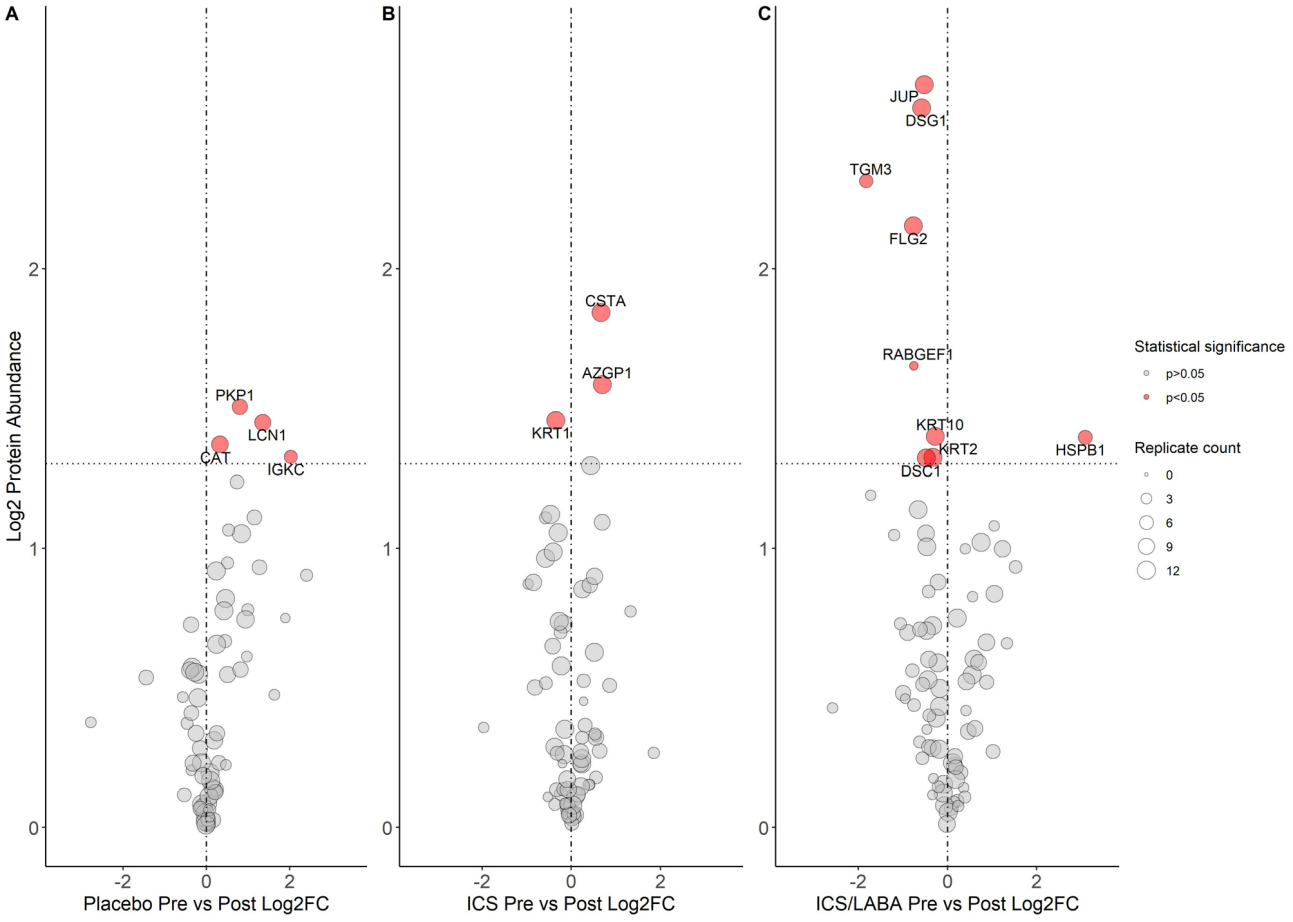
Volcano Plots demonstrating protein abundance pre- and post-RCT treatment. Log_**2**_FC: Log_2_ fold-change between groups. Vertical line represents a Log_2_FC of 0. Horizontal line is equivalent to p-value 0.05. Size of point is relative to number of samples in which protein was detected. Gene name associated with protein given if p < 0.05.

Figure 4, which includes proteins detected in all or some samples, shows significant differences before and after the three blinded inhaler treatments. Supplementary Table 3 shows the changes observed in the RCT treatment groups for proteins detected in all samples. Significant increases in abundance of DSG1 (0.58, p = 0.003), DSC1 (0.47, p = 0.048), JUP (0.52, p = 0.002), KRT2 (0.32, p = 0.047) and KRT10 (0.27, p = 0.04) occurred after ICS/LABA treatment. For proteins not detected in every sample, increases in Protein-glutamine gamma-glutamyltransferase-E (TGM3) (log_2_ fold change 1.82, p = 0.005), Filaggrin-2 (FLG2) (0.76, p = 0.007) and Rab5 GDP/GTP exchange factor (RABGEF1) (0.76, p = 0.02), and a decrease in Heat shock protein beta-1 (HSPB1) (− 3.09, p = 0.04) abundances were noted after ICS/LABA treatment. Protein network map for ICS/LABA is shown in Supplementary Fig. 4.

Following ICS treatment, significant increase in abundance of cytokeratin-1 (KRT1) (0.34, p = 0.03) and decreased abundances of cystatin-A (CSTA) (− 0.66, p = 0.01) and Zinc-alpha-2-glycoprotein (AZGP1) (− 0.70, p = 0.03) was seen. Protein network map is shown in Supplementary Fig. 5. No differences were observed for proteins detected in every sample after placebo treatment, but immunoglobulin kappa constant (IGKC) (− 2.02, p = 0.04), Lipocalin-1 (LCN1) (− 1.35, p = 0.03), Plakophilin-1 (PKP1) (− 0.80, p = 0.03) and Catalase (CAT) (− 0.33, p = 0.04) decreased, but were only noted in some samples.

**Figure 5:**
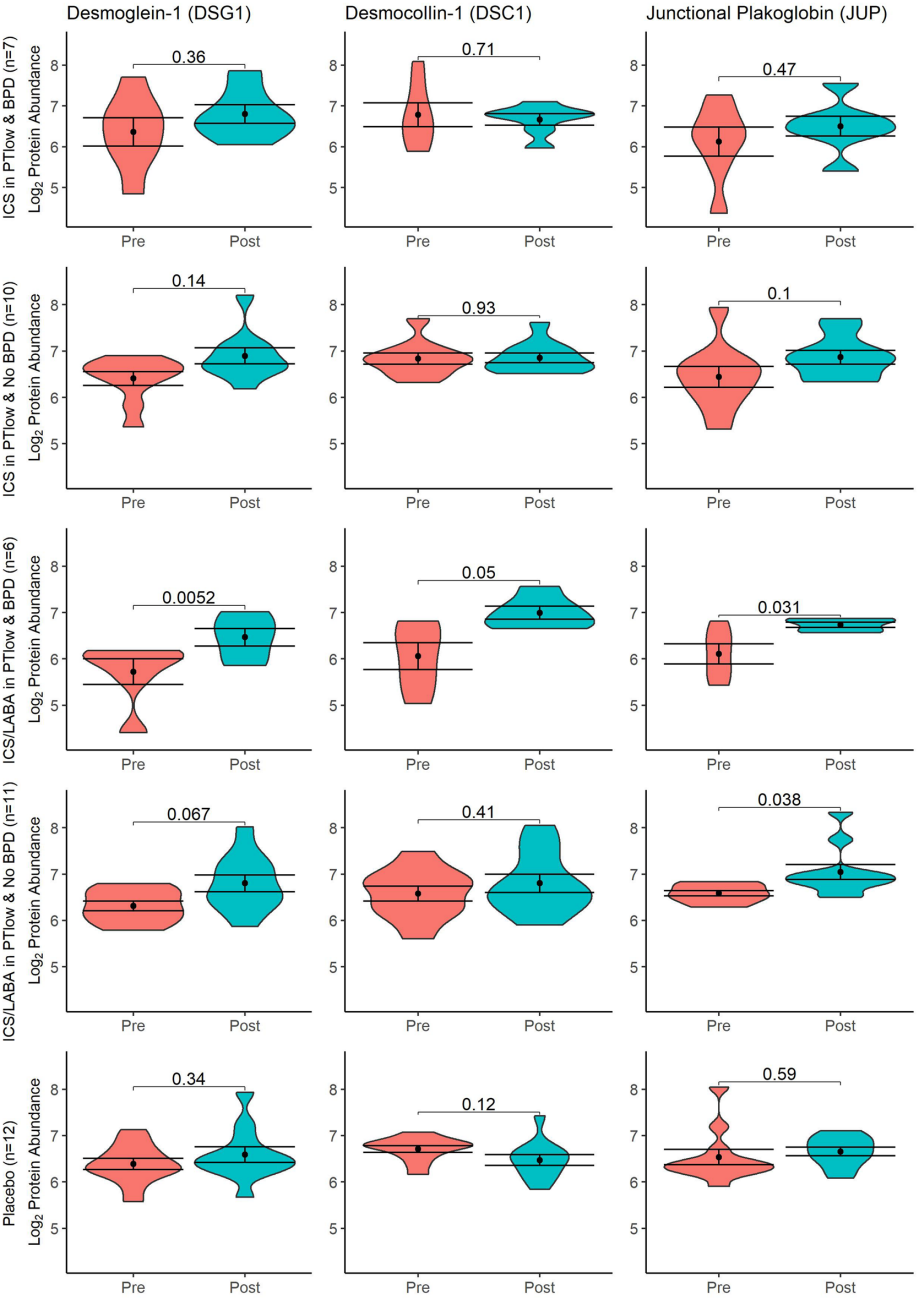
Violin plots of desmosome proteins before and after treatment with Placebo, ICS or ICS/LABA by BPD status. BPD, bronchopulmonary dysplasia; Coloured areas represent distribution of sample values. Dot and bars represent mean and standard error (SEM); Comparison bars between violin plots give p-values by paired samples t-test.

Figure 5 shows significant increases in DSG1, DSC1 and JUP occurred after ICS/LABA treatment which were not noted after ICS intervention. The PT_low_ group who had BPD in infancy had significant increases in abundance of all three proteins after ICS/LABA treatment, whereas PT_low_ without BPD only had significantly increased JUP abundance. Following ICS/LABA treatment in the PT_low_ with BPD group, levels of DSG1, DSC1 and JUP were comparable to the term control group at baseline (p = 0.56, 0.12, 0.06 respectively). Supplementary Fig. 4 demonstrates the biological links between these proteins and the changes observed for TGM3, FLG2, HSPB1, KRT2 and KRT10 as described above.

## Discussion

In this exploratory proteomic analysis, we have shown that preterm-born school-aged children who had BPD in infancy have significant differences in protein abundances for key structural proteins involving desmosomes and the cytoskeleton, several years after the initial pulmonary insult which occurred in the neonatal period. Linear regression models demonstrated that for DSG1, DSC1 and JUP, those children with a history of BPD and current low lung function had the reduced abundance of these proteins but the BPD group with normal function did not. We have recently demonstrated that ICS/LABA is an effective treatment for preterm-born children with low lung function, increasing FEV_1_ by over 14% after treatment^7^. In this study, we show that the decreases for the desmosome proteins, DSG1, DSC1 and JUP were reversed to levels observed in the term controls after 12 weeks of blinded ICS/LABA inhaler therapy. This effect was predominantly noted in children who had BPD and low lung function.

The mechanism of why some preterm-born children continue to experience lung function deficits in later life including in adulthood remains incompletely understood. Desmosomes have historically been thought to provide inert structural support to tissues, providing strong cell-to-cell adhesion; however more recent evidence shows that they have an active role in cell signalling, proliferation, migration, and apoptosis^20,21^. Despite minimal published evidence taking a proteomics approach, a reduction in desmosome proteins has been implicated in other respiratory pathologies. In a murine asthma model, bronchial wall tissue analysis noted reduced DSG1 expression following asthma exacerbation and reduced epithelial barrier integrity, potentially predisposing to further exacerbations^22^. Desmosome size and number are reduced in bronchial biopsies taken from asthmatic adults^23^, and two in-vitro studies reported that pro-inflammatory cytokines (TNF-α and IFN-γ) reduced desmosomes and JUP expression in bronchial epithelium, which was reversed by corticosteroids^24,25^.The reasons why we observed changes in DSG1, DSC1 and JUP after ICS/LABA therapy but not after ICS use is unclear. However the pathophysiology of BPD differs to that of asthma, with post-mortems of infants dying from BPD showing airway smooth muscle extension distally into peripheral airways^26^, together with peri-bronchial fibrosis and CD8+ T-lymphocyte epithelial infiltrate in adolescent survivors of BPD^27^. A recent study of adult BPD survivors has also shown a higher proportion of CD8+ cells in bronchoalveolar lavage fluid, a finding in keeping with adults with chronic obstructive pulmonary disease (COPD)^28^.

The BPD group also had increased cytokeratins (KRT6A and KRT6B). Cytokeratins comprise the intracytoplasmic cytoskeleton of epithelial tissues forming important components of intermediate filaments, which connect to desmosomes, aiding resistance to mechanical stress^29^. Although cytokeratin detection could be due to epidermal contamination, cytokeratins have previously been shown to be the most abundant proteins in EBC^30^, and both KRT6A and KRT6B have been identified as potential biomarkers for lung carcinomas in EBC proteomic analyses^13,31^. In addition, cytokeratins in EBC are potential markers of lung injury in ventilated adults^32^, and serum cytokeratin-19 fragments are increased in ventilated preterm infants who develop BPD^33^. In conjunction with the changes in DSG1, DSC1 and JUP, increased KRT6A and KRT6B in the BPD group suggest persistence of parenchymal structural abnormalities which can potentially explain the abnormal lung function observed in preterm-born subjects in childhood and adulthood.

We focused our main analyses on proteins detected in every sample to capitalise on our large sample size and ensure robust findings. Overall, the protein content of EBC was low, as previously reported^12,30^, and close to the limits of detection. Thus, we performed exploratory analyses of proteins detected only in a proportion of samples, as our methodology allowed robust quantification of these proteins in multiple replicates, most of which exceed sample sizes of many other published proteomic studies. We detected DCD in a high proportion of our samples (98%) noting significantly decreased abundance in the BPD group. DCD, a peptidase with antimicrobial activity, has been described in EBC samples previously^30^—increased detection was weakly associated with asthma in a small paediatric proteomic study^12^. In addition, we observed reduced abundance of several protease inhibitors in the BPD and PT_low_ groups, including ANXA1, SERPINB3, CSTA and SLPI, with reduced abundance of SLPI being noted in both BPD and PT_low_ groups. We have previously demonstrated protease/antiprotease imbalance, and subsequent tissue remodelling, may be implicated early in the pathogenesis of BPD^34^ but this has not been reported in later life. Tracheobronchial aspirates from ventilated preterm-born neonates who developed BPD have relative deficits of SLPI, with increased protease activity early in life^35^. Our result should be interpreted with caution as SLPI was detected in a minority of samples. ANXA1, a protease inhibitor, also known to have innate immune properties, which was decreased in the PT_low_ group but not in the BPD group, has been implicated in early lung injury in neonatal mouse models^36^, and also in proteomic studies of EBC from adults with pneumonia^31^. The decrease in antiproteases suggest an imbalance in protease/antiprotease activity, but additional work will be required in more invasive samples (e.g. bronchoalveolar lavage or induced sputum) which are ethically more challenging to obtain.

It is established that preterm born survivors, both with and without BPD, are at risk of lung function deficits in later life^3^, but there is increasing evidence that a diagnosis of BPD in infancy^16^ is a poor predictor of future lung function deficits^6,37^. In our cohort, we saw fewer differences in biologically related proteins at baseline when comparing PT_low_ and PT_c_ groups in comparison to those with and without BPD, and less than half of the children in the RCT had BPD. It is most likely that the decrease observed in DSG1, DSC1 and JUP seen in the BPD group, which is reversed by combination inhaler therapy, is due to cellular injury secondary to continuing airway inflammation^38,39^, although further work is needed to clarify this speculation. HSPB1 decreased in children treated with ICS/LABA. HSPB1 is a small heat-shock protein family member, which controls protein folding and preventing aggregation. HSPB1, which has been previously detected in the EBC proteome^40^, has been shown to have an important role in cellular responses to oxidative stress, preventing apoptosis and regulating inflammation^41^, adding further to the suggestion of chronic airway inflammation contributing to low lung function. Previous studies have also demonstrated evidence of persistent airway inflammation in children several years after preterm birth (< 32 weeks’ gestation), with increased neutrophils and IL-8 in induced sputum^38^; however, the link between this chronic inflammation and lung function parameters is not clear. We did not observe differences in proteins in the PT_low_ group who did not have BPD in infancy with the control groups; previous publications have also not been able to report a link between EBC biomarkers and lung function parameters^42^. We noted a change in protein abundances after placebo treatment which we are not able to explain. This change was not associated with any early or current life factors available to us and whether this is due to a placebo effect is speculative (especially as the lung function remained unaltered).

This study represents one of the largest proteomic analyses of EBC, and the first time, to our knowledge, that preterm-born children have been studied. By using EBC, we have been able to directly sample ALF, representative of the biochemistry of the airways, in a simple, well-tolerated and non-invasive manner. We have demonstrated that it is technically possible to perform a quantitative proteomic analysis of EBC using TMT on a large sample size and identify meaningful differences between our clinical groups. By restricting our primary analysis to proteins detected in every sample, we report robust findings, strengthened further by the modulation of these proteins of interest after inhaler treatment. Our untargeted approach and exploratory analysis of less frequently detected proteins has also implicated potentially important protease/antiprotease dynamics that future work should explore. Although collected EBC volumes varied marginally between preterm- and term-born children, our methodology was based on identifying differences in relative protein abundances thus our findings are likely to remain robust. Although some children did not complete their treatment in the RCT^7^, and some could not provide EBC after treatment, we believe that we had sufficient numbers in each group to compare EBC protein results before and after treatment. Limitations include the overall low protein content of EBC, as discussed above, and the relatively low number of proteins detected in every sample, which limited the statistical analysis approaches we could undertake. There may have been very low levels of some proteins in the samples which did not reach the limit of detection for the TMT methodology we utilised.

In conclusion, in an exploratory proteomics analysis we report significantly decreased desmosome components, DSG1, DSC1 and JUP, in children who had BPD in infancy. Furthermore, additional analyses suggest that there may be a protease/antiprotease imbalance. Taken together with the recent RCT findings^7^, these data suggest that persistent structural injury to the parenchyma in those who develop BPD in infancy is a major contributor to decreased lung function in childhood and possibly adulthood but encouragingly can be reversed by ICS/LABA treatment.

## Supplementary Information

Supplementary Information.

## Data Availability

Data from the RHiNO study is available to research collaborators subject to confidentiality and non-disclosure agreements. Contact Professor Sailesh Kotecha (kotechas@cardiff.ac.uk) for any data requests.

## References

[1] Saigal S, Doyle LW (2008). An overview of mortality and sequelae of preterm birth from infancy to adulthood. Lancet.

[2] Stoll BJ, Hansen NI, Bell EF, Walsh MC, Carlo WA, Shankaran S (2015). Trends in care practices, morbidity, and mortality of extremely preterm neonates, 1993–2012. JAMA.

[3] Kotecha SJ, Gibbons JTD, Course CW, Evans EE, Simpson SJ, Watkins WJ (2022). Geographical differences and temporal improvements in forced expiratory volume in 1 second of preterm-born children: A systematic review and meta-analysis. JAMA Pediatr..

[4] Edwards MO, Kotecha SJ, Lowe J, Richards L, Watkins WJ, Kotecha S (2016). Management of prematurity-associated wheeze and its association with atopy. PLoS ONE.

[5] Doyle LW, Irving L, Haikerwal A, Lee K, Ranganathan S, Cheong J (2019). Airway obstruction in young adults born extremely preterm or extremely low birth weight in the postsurfactant era. Thorax.

[6] Hart K, Cousins M, Watkins WJ, Kotecha SJ, Henderson AJ, Kotecha S (2022). Association of early-life factors with prematurity-associated lung disease: Prospective cohort study. Eur. Respir. J..

[7] Goulden N, Cousins M, Hart K, Jenkins A, Willetts G, Yendle L (2021). Inhaled corticosteroids alone and in combination with long-acting beta2 receptor agonists to treat reduced lung function in preterm-born children: A randomized clinical trial. JAMA Pediatr..

[8] Course CW, Kotecha S, Kotecha SJ (2019). Fractional exhaled nitric oxide in preterm-born subjects: A systematic review and meta-analysis. Pediatr. Pulmonol..

[9] Davis MD, Montpetit A, Hunt J (2012). Exhaled breath condensate: An overview. Immunol. Allergy Clin. North Am..

[10] Horvath I, Hunt J, Barnes PJ, Alving K, Antczak A, Baraldi E (2005). Exhaled breath condensate: Methodological recommendations and unresolved questions. Eur. Respir. J..

[11] Monti C, Zilocchi M, Colugnat I, Alberio T (2019). Proteomics turns functional. J. Proteom..

[12] Bloemen K, Van Den Heuvel R, Govarts E, Hooyberghs J, Nelen V, Witters E (2011). A new approach to study exhaled proteins as potential biomarkers for asthma. Clin. Exp. Allergy.

[13] Lopez-Sanchez LM, Jurado-Gamez B, Feu-Collado N, Valverde A, Canas A, Fernandez-Rueda JL (2017). Exhaled breath condensate biomarkers for the early diagnosis of lung cancer using proteomics. Am. J. Physiol0. Lung Cell Mol. Physiol..

[14] Miller MR, Hankinson J, Brusasco V, Burgos F, Casaburi R, Coates A (2005). Standardisation of spirometry. Eur. Respir. J..

[15] Quanjer PH, Stanojevic S, Cole TJ, Baur X, Hall GL, Culver BH (2012). Multi-ethnic reference values for spirometry for the 3–95-yr age range: The global lung function 2012 equations. Eur. Respir. J..

[16] Ehrenkranz RA, Walsh MC, Vohr BR, Jobe AH, Wright LL, Fanaroff AA (2005). Validation of the National Institutes of Health consensus definition of bronchopulmonary dysplasia. Pediatrics.

[17] R Core Team. R: A language and environment for statistical computing. In Version 4.0.4 ed: R Foundation for Statistical Computing, Vienna, Austria (2021).

[18] Liao Y, Wang J, Jaehnig EJ, Shi Z, Zhang B (2019). WebGestalt 2019: Gene set analysis toolkit with revamped UIs and APIs. Nucleic Acids Res..

[19] Shannon P, Markiel A, Ozier O, Baliga NS, Wang JT, Ramage D (2003). Cytoscape: A software environment for integrated models of biomolecular interaction networks. Genome Res..

[20] Holthofer B, Windoffer R, Troyanovsky S, Leube RE (2007). Structure and function of desmosomes. Int. Rev. Cytol..

[21] Green KJ, Getsios S, Troyanovsky S, Godsel LM (2010). Intercellular junction assembly, dynamics, and homeostasis. Cold Spring Harb. Perspect. Biol..

[22] Bao K, Yuan W, Zhou Y, Chen Y, Yu X, Wang X (2019). A chinese prescription Yu-Ping-Feng-San administered in remission restores bronchial epithelial barrier to inhibit house dust mite-induced asthma recurrence. Front. Pharmacol..

[23] Shahana S, Bjornsson E, Ludviksdottir D, Janson C, Nettelbladt O, Venge P (2005). Ultrastructure of bronchial biopsies from patients with allergic and non-allergic asthma. Respir. Med..

[24] Andersson K, Shebani EB, Makeeva N, Roomans GM, Servetnyk Z (2010). Corticosteroids and montelukast: Effects on airway epithelial and human umbilical vein endothelial cells. Lung.

[25] Carayol N, Campbell A, Vachier I, Mainprice B, Bousquet J, Godard P (2002). Modulation of cadherin and catenins expression by tumor necrosis factor-alpha and dexamethasone in human bronchial epithelial cells. Am. J. Respir. Cell Mol. Biol..

[26] Bush A, Busst CM, Knight WB, Hislop AA, Haworth SG, Shinebourne EA (1990). Changes in pulmonary circulation in severe bronchopulmonary dysplasia. Arch. Dis. Child..

[27] Galderisi A, Calabrese F, Fortarezza F, Abman S, Baraldi E (2019). Airway histopathology of adolescent survivors of bronchopulmonary dysplasia. J. Pediatr..

[28] Um-Bergström P, Pourbazargan M, Brundin B, Ström M, Ezerskyte M, Gao J (2022). Increased cytotoxic T-cells in the airways of adults with former bronchopulmonary dysplasia. Eur. Respir. J..

[29] Herrmann H, Bar H, Kreplak L, Strelkov SV, Aebi U (2007). Intermediate filaments: From cell architecture to nanomechanics. Nat. Rev. Mol. Cell Biol..

[30] Kurova VS, Anaev EC, Kononikhin AS, Fedorchenko KY, Popov IA, Kalupov TL (2009). Proteomics of exhaled breath: Methodological nuances and pitfalls. Clin. Chem. Lab. Med..

[31] Anaev E, Kushaeva M, Fedorchenko K, Ryabokon A, Kononikhin A, Pikin O (2017). Diagnosis of lung diseases based on proteomic analysis of exhaled breath condensate. Eur. Respir. J..

[32] Gessner C, Dihazi H, Brettschneider S, Hammerschmidt S, Kuhn H, Eschrich K (2008). Presence of cytokeratins in exhaled breath condensate of mechanical ventilated patients. Respir. Med..

[33] Panahabadi S, Heindel K, Mueller A, Holdenrieder S, Kipfmueller F (2021). Increased circulating cytokeratin 19 fragment levels in preterm neonates receiving mechanical ventilation are associated with poor outcome. Am. J. Physiol. Lung Cell Mol. Physiol..

[34] Davies PL, Spiller OB, Beeton ML, Maxwell NC, Remold-O'Donnell E, Kotecha S (2010). Relationship of proteinases and proteinase inhibitors with microbial presence in chronic lung disease of prematurity. Thorax.

[35] Watterberg KL, Carmichael DF, Gerdes JS, Werner S, Backstrom C, Murphy S (1994). Secretory leukocyte protease inhibitor and lung inflammation in developing bronchopulmonary dysplasia. J. Pediatr..

[36] Raffay TM, Locy ML, Hill CL, Jindal NS, Rogers LK, Welty SE (2013). Neonatal hyperoxic exposure persistently alters lung secretoglobins and annexin A1. Biomed. Res. Int..

[37] Corwin BK, Trembath AN, Hibbs AM (2018). Bronchopulmonary dysplasia appropriateness as a surrogate marker for long-term pulmonary outcomes: A Systematic review. J. Neonatal. Perinatal. Med..

[38] Teig N, Allali M, Rieger C, Hamelmann E (2012). Inflammatory markers in induced sputum of school children born before 32 completed weeks of gestation. J. Pediatr..

[39] Filippone M, Bonetto G, Corradi M, Frigo AC, Baraldi E (2012). Evidence of unexpected oxidative stress in airways of adolescents born very pre-term. Eur. Respir. J..

[40] Lacombe M, Marie-Desvergne C, Combes F, Kraut A, Bruley C, Vandenbrouck Y (2018). Proteomic characterization of human exhaled breath condensate. J. Breath Res..

[41] Acunzo J, Katsogiannou M, Rocchi P (2012). Small heat shock proteins HSP27 (HspB1), alphaB-crystallin (HspB5) and HSP22 (HspB8) as regulators of cell death. Int. J. Biochem. Cell Biol..

[42] Hayton C, Terrington D, Wilson AM, Chaudhuri N, Leonard C, Fowler SJ (2019). Breath biomarkers in idiopathic pulmonary fibrosis: A systematic review. Respir. Res..

