# Supplementary material for: Modulation of pulmonary desmosomes by inhaler therapy in preterm-born children with bronchopulmonary dysplasia
Source: Sci Rep. 2023 May 5;13:7330. doi: 10.1038/s41598-023-34233-5 (PMC10163267; doi:10.1038/s41598-023-34233-5)
Supplement: Supplementary file 1 — Supplementary Information. [file 41598_2023_34233_MOESM1_ESM.pdf]

Modulation of Pulmonary Desmosomes by Inhaler Therapy in Preterm-born Children with Bronchopulmonary Dysplasia – Online Supplement

Christopher W Course[1], Philip A Lewis[2], Sarah J Kotecha [1], Michael Cousins[1,3], Kylie Hart[3], W John Watkins[1], Kate J Heesom[4], Sailesh Kotecha[1]*.

[1]Department of Child Health, School of Medicine, Cardiff University, Cardiff, United Kingdom.

[2]School of Cellular and Molecular Medicine, Faculty of Life Sciences, University of Bristol, Bristol, United Kingdom

[3]Department of Paediatrics, Cardiff and Vale University Health Board, Cardiff, United Kingdom

[4]Proteomics Facility, Faculty of Life Sciences, University of Bristol, Bristol, United Kingdom

Corresponding Author: Professor Sailesh Kotecha

Department of Child Health

Cardiff University School of Medicine

Cardiff CF14 4XN

United Kingdom

kotechas@cardiff.ac.uk

Supplementary Methods:

RCT Trial:

Children received either monotherapy with inhaled corticosteroids (ICS) (50µg fluticasone propionate, two actuations taken twice a day), combination therapy of ICS and long-acting beta-agonists (LABA) (50µg fluticasone propionate and 25µg salmeterol xinafoate, two actuations taken twice a day) or placebo for twelve weeks. All trial medications were administered via a spacer device (Volumatic®, Allen & Hanburys, UK) Active trial medications were produced by St Mary's Pharmaceutical Unit, Cardiff, UK. Placebo inhalers were sourced from GlaxoSmithKline (GSK). All inhaler canisters were placed in identical plain-coloured actuators sourced from GSK to ensure adequate blinding to trial group. Participants who had previously been receiving inhaled steroid treatment prior to enrolment were weaned off steroid inhalers over a four-week washout period prior to starting the trial. All trial participants received training on good inhaler technique by the research team. To avoid ethical conflict, children receiving ICS prior to starting the trial were not randomised to the placebo arm.

Sample analysis

TMT Labelling

EBC samples were analysed at the University of Bristol Proteomics Facility. 100µL of each sample (ensuring that no sample contained more than 50µg of protein) was digested with trypsin (1.25µg trypsin; 37°C, overnight), labelled with Tandem Mass Tag (TMT) eleven plex reagents according to the manufacturer's protocol (Thermo Fisher Scientific, Loughborough, UK) and the labelled samples pooled. The pooled sample was desalted using a SepPak

cartridge according to the manufacturer's instructions (Waters, Milford, Massachusetts, USA). Eluate from the SepPak cartridge was evaporated to dryness and resuspended in 1% formic acid prior to analysis by nano-LC MSMS using an Orbitrap Fusion Lumos mass spectrometer (Thermo Scientific).

Nano-LC Mass Spectrometry

The TMT-labelled pool was fractionated using an Ultimate 3000 nano-LC system in line with an Orbitrap Fusion Lumos mass spectrometer (Thermo Scientific). In brief, peptides in 1% (vol/vol) formic acid were injected onto an Acclaim PepMap C18 nano-trap column (Thermo Scientific). After washing with 0.5% (vol/vol) acetonitrile 0.1% (vol/vol) formic acid peptides were resolved on a 250 mm × 75 μm Acclaim PepMap C18 reverse phase analytical column (Thermo Scientific) over a 150 min organic gradient, using 7 gradient segments (1-6% solvent B over 1min., 6-15% B over 58min., 15-32% B over 58min., 32-40% B over 5min., 40-90% B over 1min., held at 90% B for 6min and then reduced to 1% B over 1min.) with a flow rate of 300 nl min−1. Solvent A was 0.1% formic acid and Solvent B was aqueous 80% acetonitrile in 0.1% formic acid. Peptides were ionized by nano-electrospray ionization at 2.0kV using a stainless-steel emitter with an internal diameter of 30 μm (Thermo Scientific) and a capillary temperature of 300°C.

All spectra were acquired using an Orbitrap Fusion Lumos mass spectrometer controlled by Xcalibur 3.0 software (Thermo Scientific) and operated in data-dependent acquisition mode using an SPS-MS3 workflow. FTMS1 spectra were collected at a resolution of 120 000, with an automatic gain control (AGC) target of 200,000 and a max injection time of 50ms. Precursors were filtered with an intensity threshold of 5000, according to charge state (to

include charge states 2-7) and with monoisotopic peak determination set to Peptide. Previously interrogated precursors were excluded using a dynamic window (60s +/-10ppm). The MS2 precursors were isolated with a quadrupole isolation window of 0.7m/z. ITMS2 spectra were collected with an AGC target of 10 000, max injection time of 70ms and CID collision energy of 35%.

For FTMS3 analysis, the Orbitrap was operated at 50,000 resolution with an AGC target of 50,000 and a max injection time of 105ms. Precursors were fragmented by high energy collision dissociation (HCD) at a normalised collision energy of 60% to ensure maximal TMT reporter ion yield. Synchronous Precursor Selection (SPS) was enabled to include up to 5 MS2 fragment ions in the FTMS3 scan.

Data Analysis

The raw data files were processed and quantified using Proteome Discoverer software v2.1 (Thermo Scientific) and searched against the UniProt Human database (downloaded October 2019: 150786 entries) using the SEQUEST HT algorithm. Peptide precursor mass tolerance was set at 10ppm, and MS/MS tolerance was set at 0.6Da. Search criteria included oxidation of methionine (+15.995Da), acetylation of the protein N-terminus (+42.011Da) and Methionine loss plus acetylation of the protein N-terminus (-89.03Da) as variable modifications and carbamidomethylation of cysteine (+57.021Da) and the addition of the TMT mass tag (+229.163Da) to peptide N-termini and lysine as fixed modifications. Searches were performed with full tryptic digestion and a maximum of 2 missed cleavages were allowed. The reverse database search option was enabled, and all data was filtered to satisfy false discovery rate (FDR) of 5%.

Supplementary Table 1: List of all proteins detected within the sample, in decreasing order of number of samples in which it was detected.

UniProt Accession Number	Gene Name	Protein Name	Number of Samples	Accession Number	Gene Name	Protein Name	Number of Samples
H6VRG2	KRT1	Cytokeratin-1	218	P04040	CAT	Catalase	191
H6VRG3	KRT1	Cytokeratin-1	218	Q01469	FABP5	Fatty acid-binding protein 5	190
P01040	CSTA	Cystatin-A	218	P02768	ALB	Albumin	187
P02533	KRT14	Keratin, type I cytoskeletal 14	218	P04406	GAPDH	Glyceraldehyde-3-phosphate dehydrogenase	186
P02538	KRT6A	Keratin, type II cytoskeletal 6A	218	Q15517	CDSN	Corneodesmosin	185
P08779	KRT16	Keratin, type I cytoskeletal 16	218	Q9NZT1	CALML5	Calmodulin-like protein 5	184
P13645	KRT10	Keratin, type I cytoskeletal 10	218	P62736	ACTA2	Actin, aortic smooth muscle	165
P13647	KRT5	Keratin, type II cytoskeletal 5	218	Q7Z794	KRT77	Keratin, type II cytoskeletal 1b	165
P14923	JUP	Junction plakoglobin	218	P31151	S100A7	Protein S100-A7	164
P15924	DSP	Desmoplakin	218	P10599	TXN	Thioredoxin	156
P25311	AZGP1	Zinc-alpha-2-glycoprotein	218	P31025	LCN1	Lipocalin-1	154
P31944	CASP14	Caspase-14	218	Q13835	PKP1	Plakophilin-1	154
P35527	KRT9	Keratin, type I cytoskeletal 9	218	Q6UWP8	SBSN	Suprabasin	153
P35908	KRT2	Keratin, type II cytoskeletal 2 epidermal	218	P05089	ARG1	Arginase-1	152
P62979	RPS27A	Ubiquitin-40S ribosomal protein S27a	218	P12273	PIP	Prolactin-inducible protein	148
Q02413	DSG1	Desmoglein-1	218	Q06830	PRDX1	Peroxiredoxin-1	144
Q04695	KRT17	Keratin, type I cytoskeletal 17	218	P06702	S100A9	Protein S100-A9	142
Q08554	DSC1	Desmocollin-1	218	P60709	ACTB	Actin, cytoplasmic 1	137
Q8N1N4	KRT78	Keratin, type II cytoskeletal 78	218	Q96QE3	ATAD5	ATPase family AAA domain-containing protein 5	128
Q5D862	FLG2	Filaggrin-2	214	P32119	PRDX2	Peroxiredoxin-2	127
P81605	DCD	Dermcidin	210	A1A4E9	KRT13	Keratin 13	123
P07355	ANXA2	Annexin A2	198	Q6KB66	KRT80	Keratin, type II cytoskeletal 80	122
Q14CN4	KRT72	Keratin, type II cytoskeletal 72	198	P78386	KRT85	Keratin, type II cuticular Hb5	119
O75223	GGCT	Gamma-glutamylcyclotransferase	195	P04792	HSPB1	Heat shock protein beta-1	118
P20930	FLG	Filaggrin	194	P47929	LGALS7	Galectin-7	116
P04259	KRT6B	Keratin, type II cytoskeletal 6B	193	P05109	S100A8	Protein S100-A8	111

P01834	IGKC	Immunoglobulin kappa constant	104	Q5T749	KPRP	Keratinocyte proline-rich protein	50
Q08188	TGM3	Protein-glutamine gamma-glutamyltransferase E	102	Q5T749	KPRP	Keratinocyte proline-rich protein	50
Q13867	BLMH	Bleomycin hydrolase	101	Q9UI42	CPA4	Carboxypeptidase A4	48
Q96P63	SERPINB12	Serpin B12	99	P22531	SPRR2E	Small proline-rich protein 2E	43
B4DKJ0	Unknown	cDNA FLJ58539, highly similar to Keratin, type II cytoskeletal 4	95	Q16610	ECM1	Extracellular matrix protein 1	40
P69905	HBA1	Hemoglobin subunit alpha	93	P06733	ENO1	Alpha-enolase	39
Q86YZ3	HRNR	Hornerin	92	P08670	VIM	Vimentin	39
P0DUB6	AMY1A	Alpha-amylase 1A	91	Q9GZZ8	LACRT	Extracellular glycoprotein lacritin	35
P27482	CALML3	Calmodulin-like protein 3	88	W8QEY1	Unknown	Lactotransferrin	35
Q15323	KRT31	Keratin, type I cuticular Ha1	87	Q9UGM3	DMBT1	Deleted in malignant brain tumors 1 protein	34
Q7Z3Y8	KRT27	Keratin, type I cytoskeletal 27	84	Q9UJ41	RABGEF1	Rab5 GDP/GTP exchange factor	34
P61626	LYZ	Lysozyme C	83	O76011	KRT34	Keratin, type I cuticular Ha4	33
P02810	PRH1	Salivary acidic proline-rich phosphoprotein 1/2	81	Q9HCM4	EPB41L5	Band 4.1-like protein 5	33
P04083	ANXA1	Annexin A1	81	P62805	H4C1	Histone H4	32
Q9NSB2	KRT84	Keratin, type II cuticular Hb4	81	O76013	KRT36	Keratin, type I cuticular Ha6	31
P68871	HBB	Hemoglobin subunit beta	72	P01591	JCHAIN	Immunoglobulin J chain	30
Q14525	KRT33B	Keratin, type I cuticular Ha3-II	72	Q9C075	KRT23	Keratin, type I cytoskeletal 23	30
B2R853	Unknown	cDNA, FLJ93744, highly similar to Homo sapiens keratin 6E (KRT6E), mRNA	70	Q9Y224	RTRAF	RNA transcription, translation and transport factor protein	30
P01876	IGHA1	Immunoglobulin heavy constant alpha 1	66	P62328	TMSB4X	Thymosin beta-4	29
Q15828	CST6	Cystatin-M	66	P01857	IGHG1	Immunoglobulin heavy constant gamma 1	28
O60814	H2BC12	Histone H2B type 1-K	65	P14618	PKM	Pyruvate kinase PKM	28
P00441	SOD1	Superoxide dismutase	61	Q8TF66	LRRC15	Leucine-rich repeat-containing protein 15	28
P23490	LORICRIN	Loricrin	61	G3V1M9	PRB1	Basic salivary proline-rich protein 1	27
P29508	SERPINB3	Serpin B3	58	P12035	KRT3	Keratin, type II cytoskeletal 3	27
A0A0S2Z4G4	TPM3	Tropomyosin 3 isoform 1	57	P68431	H3C1	Histone H3.1	27
Q96DR8	MUCL1	Mucin-like protein 1	54	Q9BYR6	KRTAP3-3	Keratin-associated protein 3-3	25
O43790	KRT86	Keratin, type II cuticular Hb6	53	Q9HCY8	S100A14	Protein S100-A14	25

P22735	TGM1	Protein-glutamine gamma-glutamyltransferase K	24	Q92820	GGH	Gamma-glutamyl hydrolase	15
P82279	CRB1	Protein crumbs homolog 1	24	Q9UKX2	MYH2	Myosin-2	14
Q05639	EEF1A2	Elongation factor 1-alpha 2	23	P07339	CTSD	Cathepsin D	13
P04080	CSTB	Cystatin-B	20	P78385	KRT83	Keratin, type II cuticular Hb3	13
P15090	FABP4	Fatty acid-binding protein, adipocyte	20	P31947	SFN	14-3-3 protein sigma	12
P63104	YWHAZ	14-3-3 protein zeta/delta	20	P60174	TPI1	Triosephosphate isomerase	12
Q52LG2	KRTAP13-2	Keratin-associated protein 13-2	20	P42357	HAL	Histidine ammonia-lyase	11
Q5T750	XP32	Skin-specific protein 32	20	P02814	SMR3B	Submaxillary gland androgen-regulated protein 3B	10
Q6ZVX7	NCCRP1	F-box only protein 50	20	P0DPA2	VSIG8	V-set and immunoglobulin domain-containing protein 8	10
P35579	MYH9	Myosin-9	19	P16615	ATP2A2	Sarcoplasmic/endoplasmic reticulum calcium ATPase 2	10
Q6E0U4	DMKN	Dermokine	19	Q6A163	KRT39	Keratin, type I cytoskeletal 39	10
Q8IUC0	KRTAP13-1	Keratin-associated protein 13-1	19	Q96DA0	ZG16B	Zymogen granule protein 16 homolog B	10
B4DJM5	Unknown	cDNA FLJ61294, highly similar to Keratin, type I cytoskeletal 17	18	Q9NQ38	SPINK5	Serine protease inhibitor Kazal-type 5	10
P01036	CST4	Cystatin-S	18	Q9UKK9	NUDT5	ADP-sugar pyrophosphatase	10
P02452	COL1A1	Collagen alpha-1(I) chain	18	A2IDD5	CCDC78	Coiled-coil domain-containing protein 78	9
P03973	SLPI	Secretory leukocyte peptidase inhibitor	18	P02812	PRB2	Basic salivary proline-rich protein 2	9
Q14533	KRT81	Keratin, type II cuticular Hb1	18	P06454	PTMA	Prothymosin alpha	9
Q3LI77	KRTAP13-4	Keratin-associated protein 13-4	18	P16104	H2AX	Histone H2AX	9
Q96QA5	GSDMA	Gasdermin-A	18	Q02383	SEMG2	Semenogelin-2	9
P13929	ENO3	Beta-enolase	17	Q3SY84	KRT71	Keratin, type II cytoskeletal 71	9
Q14574	DSC3	Desmocollin-3	17	Q6S8J3	POTEE	POTE ankyrin domain family member E	9
Q2PPJ7	RALGAPA2	RalGTPase-activating protein subunitalpha-2	17	Q6YFL4	KRTHB6	Type II keratin (Fragment)	9
Q6ZUA9	MROH5	Maestro heat-like repeat family member 5	17	Q92764	KRT35	Keratin, type I cuticular Ha5	9
P17066	HSPA6	Heat shock 70 kDa protein 6	15	Q9BZE2	PUS3	tRNA pseudouridine(38/39) synthase	9
P22528	SPRR1B	Cornifin-B	15	Q9HC84	MUC5B	Mucin-5B (MUC-5B)	9
Q14508	WFDC2	WAP four-disulfide core domain protein 2	15	P04279	SEMG1	Semenogelin-1	8

P05976	MYL1	Myosin light chain 1/3, skeletal muscle isoform	8	Q9BYR9	KRTAP2-4	Keratin-associated protein 2-4	7
P09228	CST2	Cystatin-SA	8	O43829	ZBTB14	Zinc finger and BTB domain-containing protein 14	6
P11055	MYH3	Myosin-3	8	Q5XKE5	KRT79	Keratin, type II cytoskeletal 79	6
P31949	S100A11	Protein S100-A11	8	Q9Y618	NCOR2	Nuclear receptor corepressor 2	6
P49454	CENPF	Centromere protein F	8	P13639	EEF2	Elongation factor 2	5
P52566	ARHGDIB	Rho GDP-dissociation inhibitor 2	8	P62937	PPIA	Peptidyl-prolyl cis-trans isomerase A	5
P00491	PNP	Purine nucleoside phosphorylase	7	A0A1B0GVI3	KRT10	Keratin, type I cytoskeletal 10 (Fragment)	4
P09211	GSTP1	Glutathione S-transferase P	7	D3DTX7	COL1A1	Collagen, type I, alpha 1, isoform CRA_a	4
P11021	HSPA5	Endoplasmic reticulum chaperone BiP	7	O95678	KRT75	Keratin, type II cytoskeletal 75	4
P47756	CAPZB	F-actin-capping protein subunit beta	7	P05455	SSB	Lupus La protein	4
P60900	PSMA6	Proteasome subunit alpha type-6	7	Q9BYR8	KRTAP3-1	Keratin-associated protein 3-1	4
Q8NEZ4	KMT2C	Histone-lysine N-methyltransferase 2C	7	Q9H1E1	RNASE7	Ribonuclease 7	4
Q8WVV4	POF1B	Protein POF1B	7	Q15149	PLEC	Plectin	2

Supplementary Table 2: Detected proteins with a significant abundance difference between groups.

UniProt Accession Number	Gene Name	Protein Name	Protein Function	Preterm vs Term n = 149 v 69				BPD vs No BPD n = 37 v 112				PT_{low} vs PT_c n = 53 v 97			
				n	Log_2FC	Ratio	p	n	Log_2FC	Ratio	p	n	Log_2FC	Ratio	p
P81605	DCD	Dermcidin	Peptidase Antimicrobial activity	146 v 64	-0.03	0.98	0.83	**37 v 109**	**-0.42**	**0.75**	**0.03***	52 v 94	0.23	1.17	0.16
P04259	KRT6B	Keratin, type II cytoskeletal 6B	Cytoskeleton	**133 v 60**	**0.54**	**1.45**	**0.03***	**33 v 100**	**0.76**	**1.69**	**0.03***	46 v 87	0.34	1.27	0.25
Q01469	FABP5	Fatty acid-binding protein 5	Lipid transporter	122 v 68	-0.19	0.88	0.27	27 v 95	0.06	1.04	0.77	**38 v 84**	**-0.39**	**0.76**	**0.04***
P62736	ACTA2	Actin, aortic smooth muscle	Muscle protein	**115 v 50**	**0.54**	**1.45**	**0.01***	31 v 84	-0.43	0.74	0.10	46 v 69	-0.07	0.95	0.77
P05089	ARG1	Arginase-1	Hydrolase Antimicrobial activity	104 v 48	-0.03	0.98	0.86	29 v 75	-0.25	0.84	0.17	**37 v 67**	**-0.39**	**0.76**	**0.03***
Q6UWP8	SBSN	Suprabasin	Unknown	**99 v 54**	**-0.33**	**0.80**	**0.02***	**25 v 74**	**-0.34**	**0.79**	**0.049***	36 v 63	-0.12	0.92	0.43
Q6KB66	KRT80	Keratin, type II cytoskeletal 80	Cytoskeleton	**72 v 50**	**-0.27**	**0.83**	**0.01***	18 v 54	0.09	1.06	0.52	24 v 48	0.02	1.01	0.89
P04083	ANXA1	Annexin A1	Protease inhibitor Anti-inflammatory activity	50 v 31	-0.09	0.94	0.75	14 v 36	0.19	1.14	0.57	**15 v 35**	**-0.77**	**0.59**	**0.02***
P23490	LORICRIN	Loricrin	Cytoskeleton	**42 v 19**	**-0.42**	**0.75**	**0.03***	10 v 32	-0.08	0.95	0.67	14 v 28	-0.23	0.85	0.20
P29508	SERPINB3	Serpin B3	Protease inhibitor	37 v 21	0.59	1.51	0.19	12 v 25	0.23	1.17	0.62	**12 v 25**	**-0.86**	**0.55**	**0.01***
P22531	SPRR2E	Small proline-rich protein 2E	Peptide cross-linking	36 v 7	0.07	1.05	0.88	**12 v 24**	**-0.92**	**0.53**	**0.04***	16 v 20	-0.40	0.76	0.33
P06733	ENO1	Alpha-enolase	Transcription regulation	**29 v 10**	**-0.64**	**0.64**	**0.01***	6 v 23	0.52	1.43	0.19	7 v 22	0.36	1.28	0.36
Q9HCM4	EPB41L5	Band 4.1-like protein 5	Cytoskeleton	22 v 11	-0.38	0.77	0.35	**4 v 18**	**0.68**	**1.60**	**0.04***	7 v 15	0.61	1.53	0.21
Q9C075	KRT23	Keratin, type I cytoskeletal 23	Cytoskeleton	**22 v 8**	**-1.19**	**0.44**	**0.01***	6 v 16	-0.05	0.97	0.91	7 v 15	0.51	1.42	0.35
P22735	TGM1	Protein-glutamine gamma-glutamyltransferase K	Acyltransferase Peptide cross-linking	**15 v 9**	**-0.62**	**0.65**	**0.047***	4 v 11	-0.56	0.68	0.36	5 v 10	0.32	1.25	0.55

P63104	YWHAZ	14-3-3 protein zeta/delta	Signalling regulator	**11 v 9**	**-0.70**	**0.62**	**0.04***	3 v 8	0.05	1.04	0.88	2 v 9	-0.87	0.55	0.47
P01036	CST4	Cystatin-S	Protease inhibitor	13 v 5	-0.23	0.85	0.79	**5 v 8**	**-1.23**	**0.43**	**0.004***	6 v 7	-0.29	0.82	0.58
P03973	SLPI	Secretory leukocyte antipeptidase inhibitor	Protease inhibitor Antimicrobial activity	11 v 7	0.99	1.99	0.18	**2 v 9**	**-2.08**	**0.24**	**0.04***	**2 v 9**	**-2.32**	**0.20**	**0.03***
Q96QA5	GSDMA	Gasdermin-A	Pore-forming protein	**11 v 7**	**1.03**	**2.04**	**0.02***	4 v 7	-0.06	0.96	0.93	6 v 5	-0.92	0.53	0.13
Q6ZUA9	MROH5	Maestro heat-like repeat family member 5	Unknown	6 v 11	0.01	1.01	0.99	2 v 4	0.18	1.13	0.82	**3 v 3**	**-1.48**	**0.36**	**0.01***
Q92820	GGH	Gamma-glutamyl hydrolase	Peptidase	12 v 3	-0.66	0.63	0.30	**2 v 10**	**-0.79**	**0.58**	**0.03***	2 v 10	0.08	1.06	0.81
Q9BZE2	PUS3	tRNA pseudouridine(38/39) synthase	Isomerase	**7 v 2**	**-1.66**	**0.32**	**0.004***	**2 v 5**	**1.66**	**3.16**	**0.004***	2 v 5	0.03	1.02	0.98
P60900	PSMA6	Proteasome subunit alpha type-6	Peptidase	7 v 0	NA	NA	NA	2 v 5	-0.35	0.78	0.37	**2 v 5**	**1.05**	**2.07**	**0.02***

BPD: Preterm-born with history of bronchopulmonary dysplasia; PT_c: Preterm-born control; PT_{low}: Preterm born with low lung function; Log_2FC: Log_2 fold-change between groupings

*Denotes p-vale <0.05 from Welch's t-test

Supplementary Table 3: Treatment effect on proteins detected in every sample.

UniProt Accession Number	Gene Name	Protein Name	Protein Function	Placebo n=12 Pre vs Post			ICS n=17 Pre vs Post			ICS/LABA n=17 Pre vs Post		
				Log_2FC	Ratio	p	Log_2FC	Ratio	p	Log_2FC	Ratio	p
Q08554	DSC1	Desmocollin-1	Cell-cell junction	0.24	1.18	0.12	0.04	1.03	0.83	**-0.47**	**0.72**	**0.048***
Q02413	DSG1	Desmoglein-1	Cell-cell junction	-0.20	0.87	0.34	-0.47	0.72	0.08	**-0.58**	**0.67**	**0.003***
P15924	DSP	Desmoplakin	Cell-cell junction, Cytoskeleton	0.07	1.05	0.77	-0.18	0.88	0.19	-0.21	0.86	0.26
P14923	JUP	Junction plakoglobin	Plasma membrane protein complex	-0.12	0.92	0.59	-0.41	0.75	0.10	**-0.52**	**0.70**	**0.002***
H6VRG2	KRT1	Cytokeratin-1	Cytoskeleton	-0.18	0.88	0.28	**-0.34**	**0.79**	**0.03***	-0.17	0.89	0.32
H6VRG3	KRT1	Cytokeratin-1	Cytoskeleton	-0.34	0.79	0.27	-0.58	1.49	0.11	-0.33	0.80	0.19
P35908	KRT2	Keratin, type II cytoskeletal 2 epidermal	Cytoskeleton	0.10	1.07	0.68	-0.16	0.90	0.55	**-0.32**	**0.80**	**0.047***
P13647	KRT5	Keratin, type II cytoskeletal 5	Cytoskeleton	-0.03	0.98	0.89	0.02	1.01	0.92	0.02	1.01	0.88
P02538	KRT6A	Keratin, type II cytoskeletal 6A	Cytoskeleton	0.19	1.14	0.73	0.24	1.18	0.59	0.60	1.52	0.25
P35527	KRT9	Keratin, type I cytoskeletal 9	Cytoskeleton	-0.28	0.82	0.28	-0.30	0.81	0.09	0.11	1.08	0.59
P13645	KRT10	Keratin, type I cytoskeletal 10	Structural protein extracellular space	0.09	1.06	0.64	-0.14	0.91	0.45	**-0.27**	**0.83**	**0.04***
P02533	KRT14	Keratin, type I cytoskeletal 14	Cytoskeleton	0.03	1.02	0.82	-0.22	1.16	0.26	0.22	1.16	0.18
P08779	KRT16	Keratin, type I cytoskeletal 16	Cytoskeleton	-0.11	0.93	0.83	0.07	1.05	0.90	0.55	1.46	0.28
Q04695	KRT17	Keratin, type I cytoskeletal 17	Intermediate filament cytoskeleton	-0.01	0.99	0.98	0.24	1.18	0.57	0.76	1.69	0.10
Q8N1N4	KRT78	Keratin, type II cytoskeletal 78	Cytoskeleton	0.24	1.18	0.22	-0.27	0.83	0.18	-0.17	0.89	0.37
P31944	CASP14	Caspase-14	Protease	0.85	1.80	0.09	0.52	1.43	0.24	-0.44	0.74	0.30
P01040	CSTA	Cystatin-A	Protease inhibitor	-0.07	0.95	0.85	**0.66**	**1.58**	**0.01***	-0.25	0.84	0.41
P62979	RPS27A	Ubiquitin-40S ribosomal protein S27a	Structural component of ribosome	-0.02	0.99	0.95	0.44	1.36	0.05	-0.18	0.88	0.52
P25311	AZGP1	Zinc-alpha-2-glycoprotein	Major histocompatibility complex protein	0.46	1.38	0.15	**0.70**	**1.62**	**0.03***	-0.10	0.93	0.75

ICS: Inhaled corticosteroids; ICS/LABA: Inhaled corticosteroids/long-acting beta agonists; Log_2FC: Log_2 fold-change between groups. *Denotes p-vale <0.05 from paired samples t-test

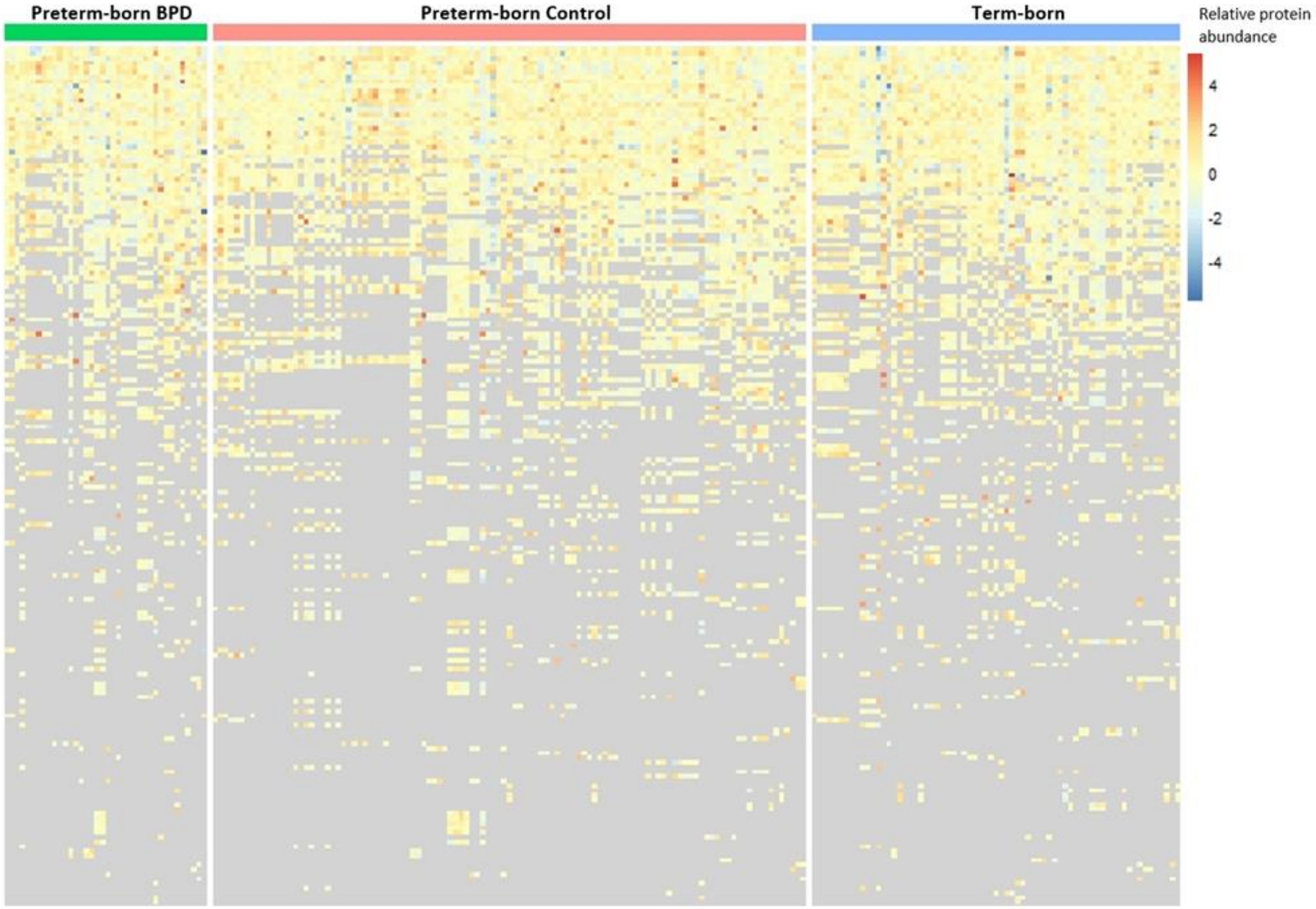

Supplementary Figure 1: Heatmap displaying Protein Content of Baseline EBC Samples. Individual samples represented in columns. Coloured areas represent relative protein abundance. Grey areas represent proteins not detected within that sample.

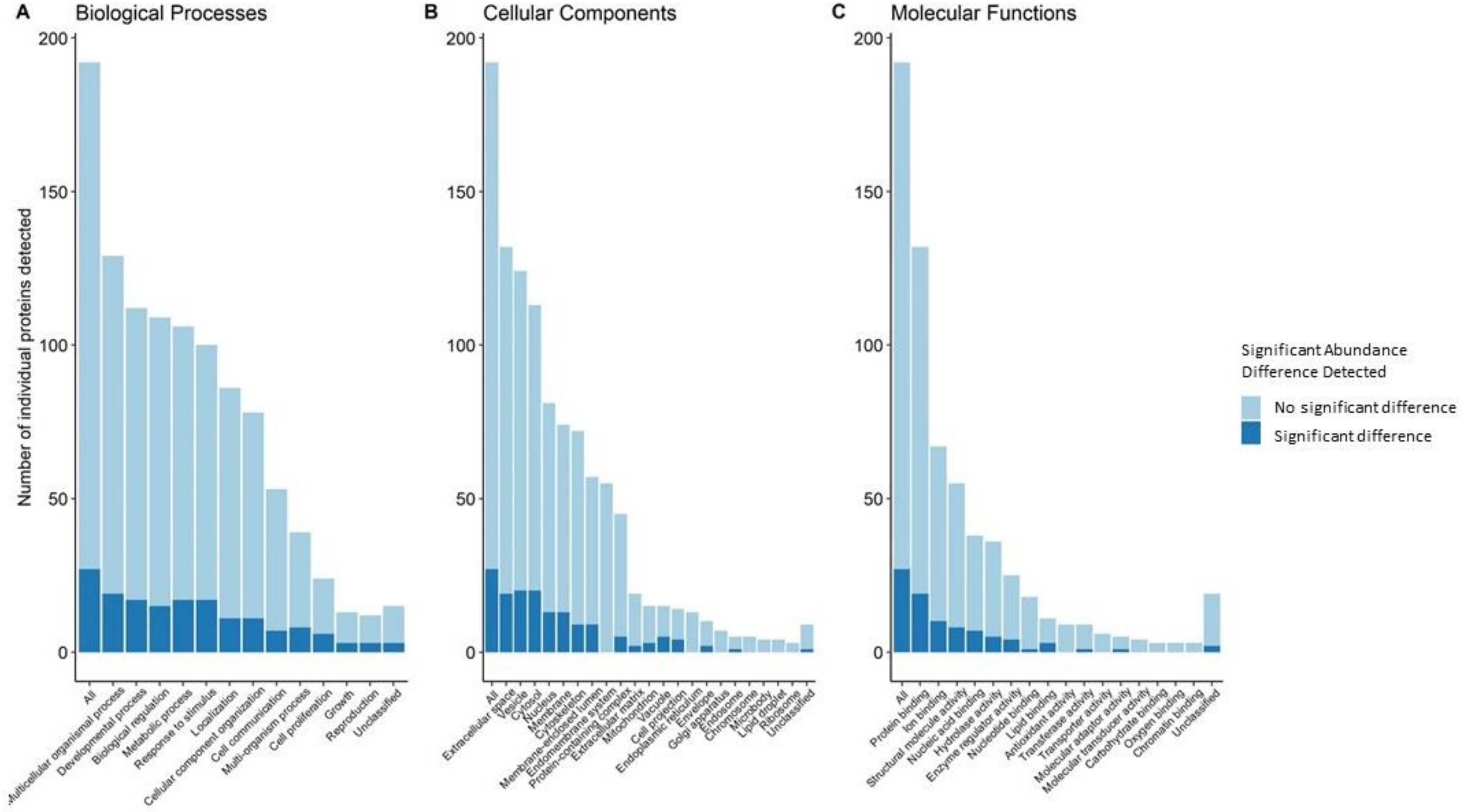

Supplementary Figure 2: Functional enrichment analysis of proteins with and without significant abundance difference between clinical groups.

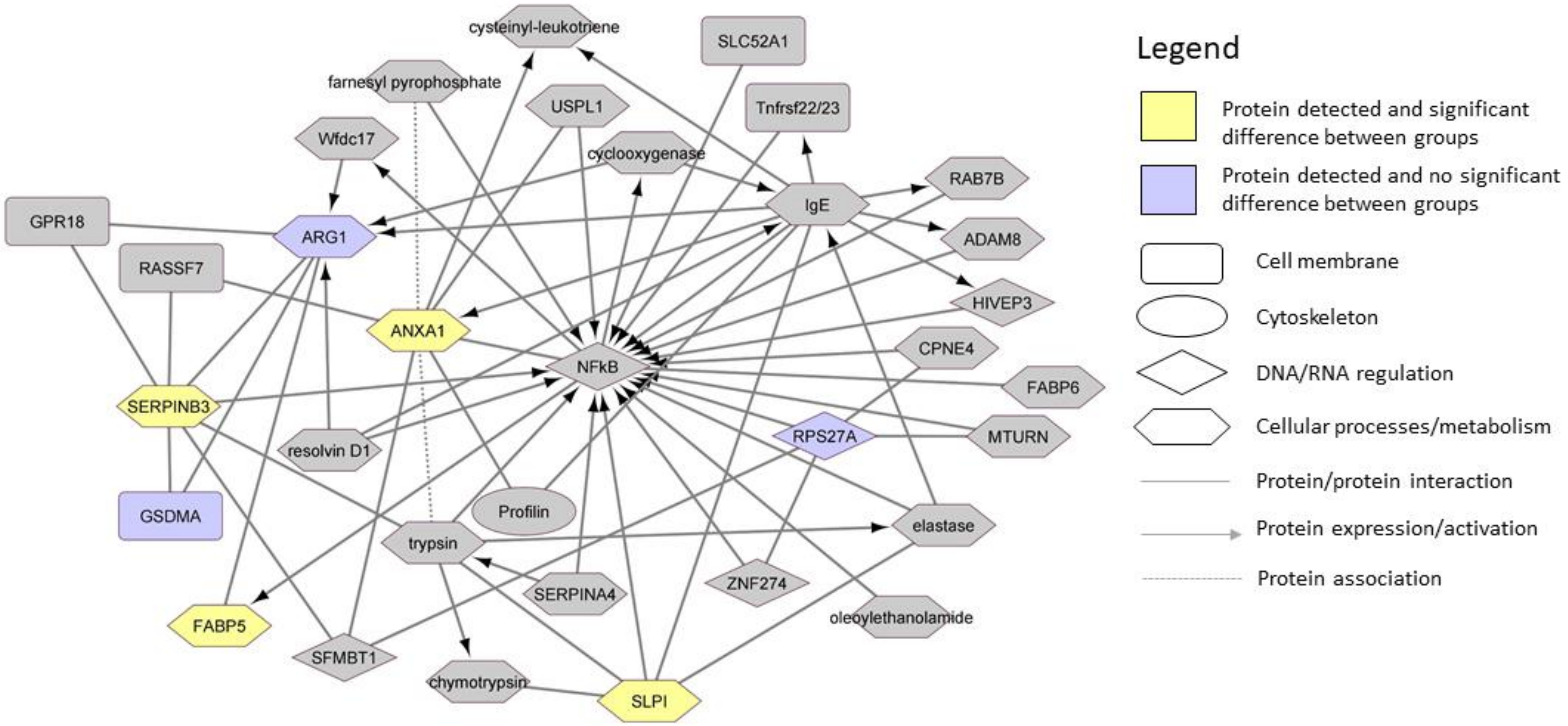

Supplementary Figure 3: Protein network map of significant protein differences between PT_{low} and PT_c groups.

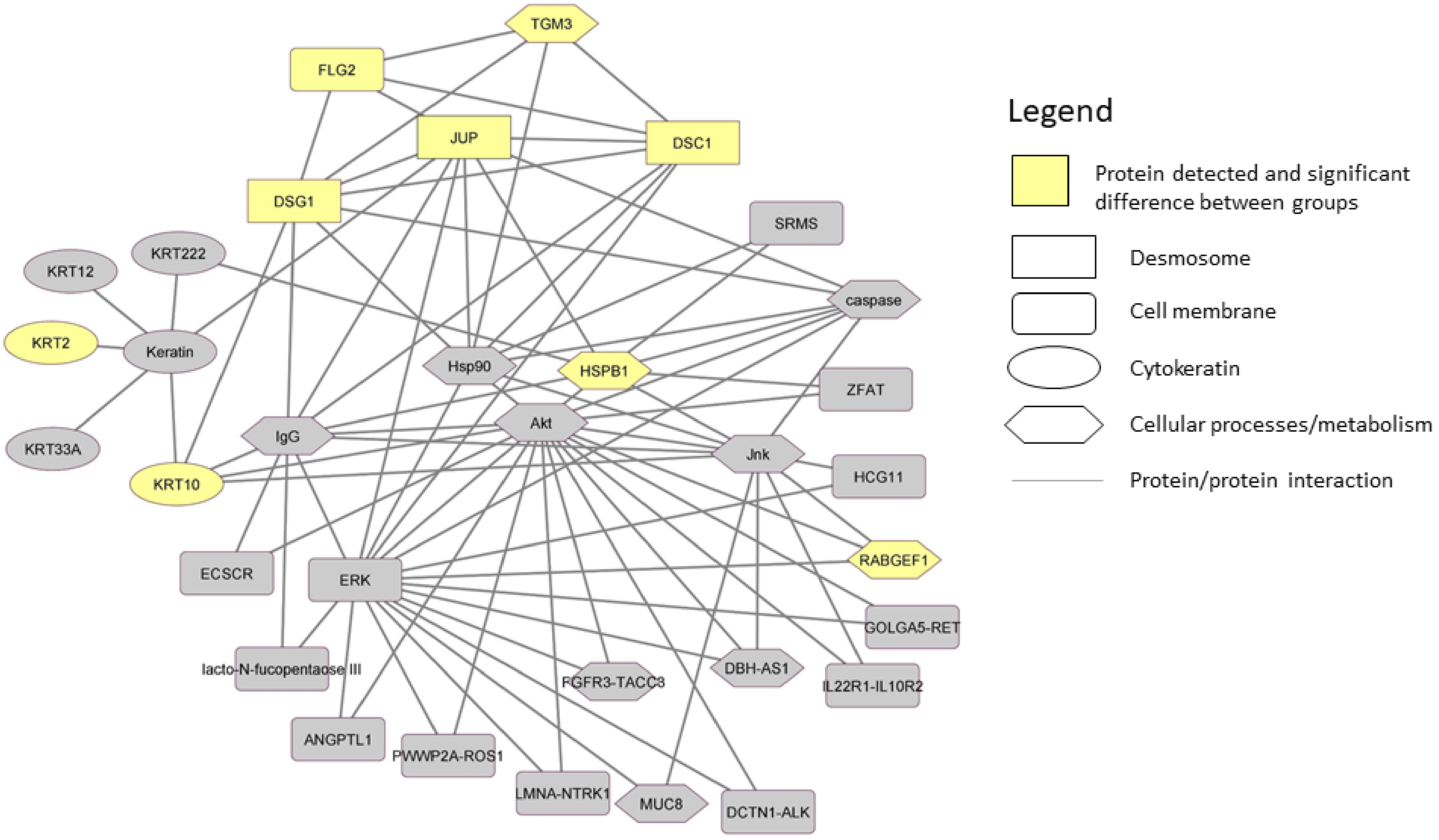

Supplementary Figure 4: Protein network map of significant protein differences before and after ICS/LABA treatment.

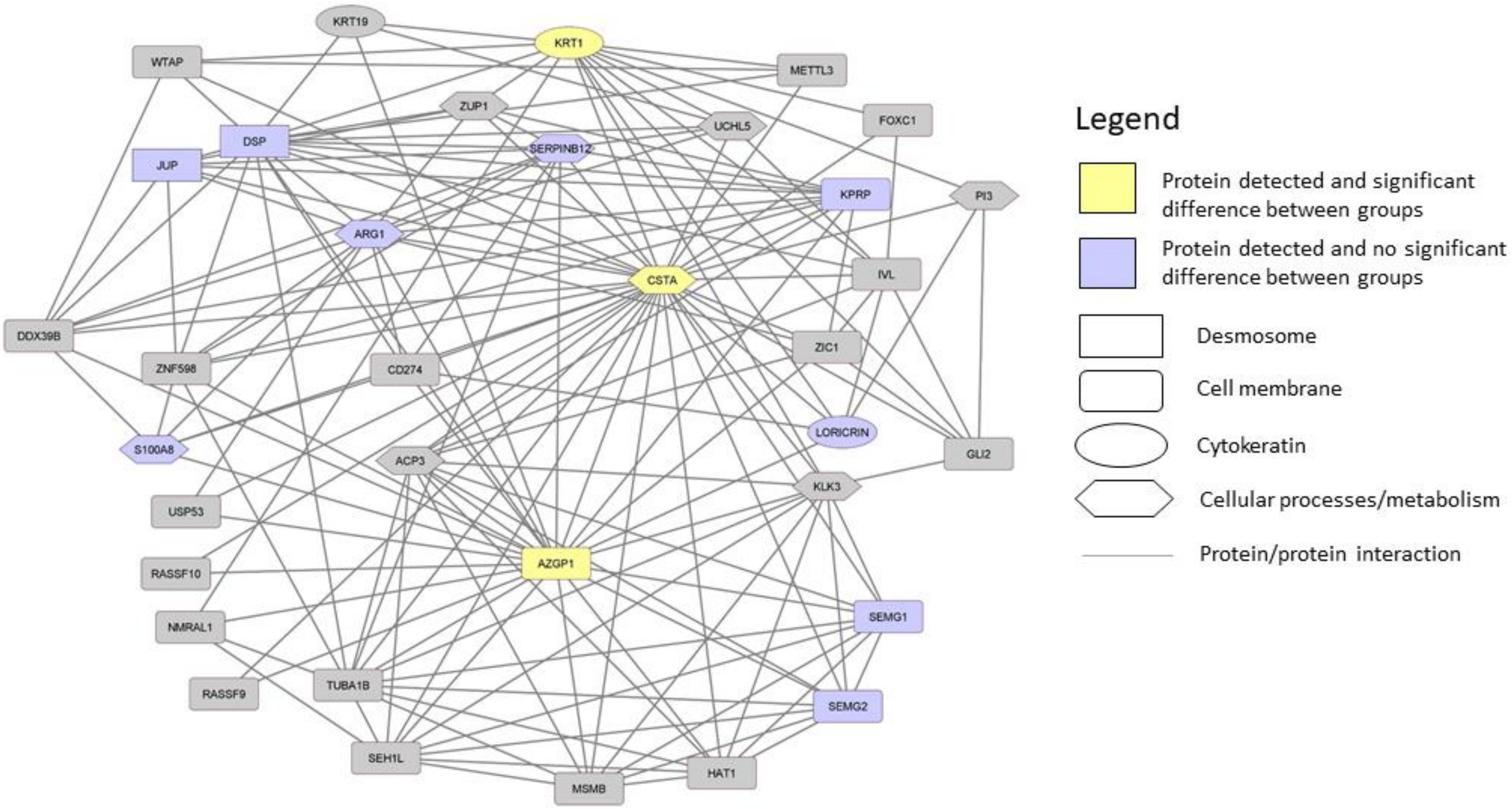

Supplementary Figure 5: Protein network map of significant protein differences before and after ICS treatment.
